# Bottlenecks Analysis in the Intervention of Improving Maternal Health in Rural Areas of Tanzania: A Convergent Mixed-Method Approach

**DOI:** 10.34172/ijhpm.8355

**Published:** 2025-03-02

**Authors:** Hyeyun Kim, Jiye Kim, Seohyeon Lee, Minkang Cho, Hyekyeong Kim

**Affiliations:** ^1^Korea Institute for Health and Social Affairs, Sejong, Republic of Korea.; ^2^Department of Health Convergence, Graduate School of Ewha Womans University, Seoul, Republic of Korea.; ^3^Department of Health Convergence, Ewha Womans University, Seoul, Republic of Korea.

**Keywords:** Bottleneck, Maternal Health, Intervention, Mixed-Method

## Abstract

**Background:**

Achieving universal health coverage for maternal health (MH) requires a health system that ensures the availability, accessibility, acceptability, and effective use of services. The study aimed to identify bottlenecks that hinder project outcomes of MH in the rural districts of Tanzania.

**Methods:**

This study employed a convergent mixed-method approach to conduct the bottleneck analysis. Quantitative data were collected to identify indicators of MH utilization, with source including Tanzanian health statistics, health facilities and the women in reproductive age (WRA) survey. In-depth interviews (IDIs) and focus group discussions (FGDs) were conducted with WRA, their families, community health workers (CHWs), and healthcare personnel (HP) to gain insight into factors influencing healthcare utilization from both a demand and an environmental perspective. Following the Tanahashi steps, the quantitative data were analyzed using descriptive statistics and the qualitative data were analyzed using a thematic approach. The findings from both were integrated to identify bottlenecks toward effective coverage and how bottlenecks affected the utilization of MH services.

**Results:**

Community awareness and acceptance were observed to be high, however only a limited number of individuals had received MH services. Utilization rates for antenatal care (ANC) and postnatal care (PNC) were 17.4% and 22.0%, respectively. This suggests that efforts to enhance awareness may be inadequate to change social norms and lead to health behaviors. Furthermore, even when women utilize the service, they may not do so in a timely or consistent manner due to low service quality or unsatisfactory experiences.

**Conclusion:**

To strengthen the logic model, contextual factors such as provider attitudes, service quality, supportive family, and community climate need to be considered to ensure that WRAs are satisfied with and continue to access services. With building supply-side infrastructure, ongoing efforts to change stakeholders’ perceptions of MH services and utilization patterns will be needed to improve the coverage of MH services.

## Background

**Key Messages**

**Implications for policy makers**

- Policy-makers should consider the possibility that contextual factors not included in the logic model of maternal health (MH) programs may act as cumulative barriers and affect health outcomes.
- While the provision of healthcare is of significant importance, it is also essential to enhance the quality of healthcare by implementing feedback mechanisms for negative experiences at healthcare facilities.
- It is recommended that awareness-raising and knowledge-building activities, such as photovoice and community seminars, be conducted on a regular and consistent basis to facilitate the actual utilization of MH services among community members.

**Implications for the public**

Providing appropriate maternal health (MH) services to pregnant women requires well equipped health facilities, health workers, and equipment to deliver services, and the quality of services is also important to ensure satisfaction with the healthcare experience. Interventions are needed to increase pregnant women’s knowledge of MH and their willingness to use services, and it should be sufficiently targeted to translate into actual use of health services. Acceptance of the need for MH care at the community level is necessary for pregnant women to continue seeking MH services, as husbands or mothers-in-law have greater decision-making power than women and social norms strongly influence health service utilization in Tanzania.

Maternal health (MH) services are a fundamental component of primary healthcare, particularly in developing countries where maternal mortality rates remain high. Despite global initiatives to enhance accessibility to these services, considerable obstacles to the demand and supply of MH services in developing countries persisit.^1-3^ According to the Ministry of Health and Social Welfare of Tanzania over 90% of the population lived within 5 km of a health facility. However, these facilities are not functioning effectively due to outdated infrastructure and poor service quality.Furthermore, Tanzania also faced a significant shortage of health personnel, largely due to a lack of medical graduates, inadequate remuneration, and poor working conditions.^4-7^

 In order to achieve universal health coverage for MH, it is necessary for a health system to ensure the availability, easy accessibility, acceptability, and effective utilization of existing MH services.^8^ This can be achieved through the sustainable performance of development cooperation projects, which require the project to be planned based on a logical framework comprising inputs, activities, outputs, outcomes, and goals.^2,9^ The launch of the Sustainable Development Goals has also led to an increased emphasis on the continued effectiveness of Official Development Assistance projects.^10^ To avoid the point at which a gap in the logic model between project inputs, activities, outputs, and outcomes occurs, it is essential to identify the factors that influence pregnant women’s utilization of MH services. Previous studies evaluating MH programs in developing countries have shown that both supply-side and demand-side factors affect access to MH services.

 On the demand-side, socioeconomic factors such as poverty, limited knowledge of MH, and cultural practices may discourage the use of MH services. Furthermore, a lack of trust in healthcare providers may act as an additional barrier.^5,11,12^ A further differentiating factor in the context of MH services in developing countries is the greater decision-making power of families regarding access to these services. A higher proportion of women accessed antenatal care (ANC) when they made the decision by herself, as opposed to when their husbands were the decision-maker. However, women are constrained in their capacity to autonomously determine whether to use or benefit from MH services.^13^ While previous studies have suggested factors that influence access to MH services, it is unclear how these factors operate at different stages of service utilization and how they affect actual utilization and outcomes. The objective of the study was to identify the primary bottlenecks that hinder the achievement of project outcomes through a process evaluation. Moreover, this study aimed to ascertain which components of the logic model were associated with the identified bottlenecks. The study sought to recommend strategies for enhancing the effectiveness of MH services through optimization of the project’s logic model.

## Methods

###  Setting of Maternal Health Project in Tanzania

 This study was a process evaluation of the Maternal Health Improvement Project in Kishapu, Tanzania. The project was designed to benefit pregnant women and their families residing in Kishapu, a region with limited access to medical care. Following training, healthcare personnel (HP) and community health workers (CHWs) were deployed to provide maternal and newborn health (MNH) services. The project encompassed a number of key objectives, including the reinforcement of the capacity to provide essential MNH services, the improvement of the service-related infrastructure, such as equipment and medicines, in healthcare facilities, the expansion of access to these services, the establishment of a program designed to enhance awareness of MH within the community, and the promotion of organized advocacy for the health of these vulnerable groups.

**Figure 1:**
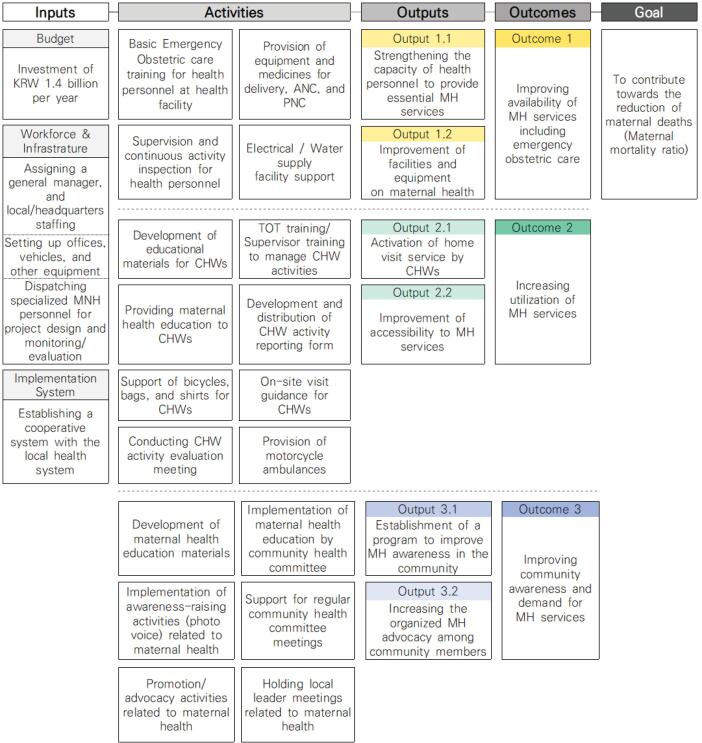
Logic Model of Maternal Health Project in Tanzania.Abbreviations: CHW, community health worker; MH, maternal health; ANC, antenatal care; PNC, postnatal care; TOT, training of trainers; MNH, maternal and newborn health; KRW, Korean Won.

 The US Agency for International Development developed the Logical Framework Approach to ensure neutrality and transparency of aid, which is widely adopted by many aid organizations for project planning.^14,15^ It constructs a logic model of goal, outcomes, outputs activities, and inputs that are aligned with the planning, implementation, monitoring, and evaluation of a project. The logic model of the project is shown in (Figure 1).

###  Theoretical Model and Study Design

 This study applied a bottleneck analysis based on the Tanahashi model to gain a more systematic understanding of the point at which a “gap” occurs in the derivation of the logical link between inputs, activities, outputs, and outcomes. In 1978, Tanahashi outlined a methodology for measuring health service coverage and identifying bottlenecks in the allocation of resources to achieve desired health outcomes.^16^ This approach is useful for assessing equity in service coverage and identifying gaps to target interventions for vulnerable populations.^8,17^ The United Nations Children’s Fundhas consistently applied Tanahashi model to the evaluation of MNH projects in low- and middle-income countries until recently.^18^

 The model typically comprises five stages of healthcare service delivery, which collectively constitute the implementation pathway. The five stages of healthcare service delivery are: availability, accessibility, acceptability, utilization, and effectiveness coverage.^16^ In the field of healthcare, the utilization of healthcare services can only be achieved if health facilities and personnel are provided (availability), if services are accessible both geographically and economically (accessibility), and if the services are socially and culturally acceptable to the user (acceptability). It is only through the achievement of both effective coverage (effectiveness) and contact coverage (initial utilization) of healthcare services that improvements in health outcomes can be expected. The Tanahashi model can be modified according to specific requirements of the evaluation in question.^2,17^ Based on the criteria proposed by Baker et al,^2^ a bottleneck was defined as a difference of 30%p or more from the value in the previous step. In this study, the term “bottleneck” was also defined as a decrease of more than half from the value in the previous step.

 In this study, the bottleneck analysis was conducted through a convergent mixed-methods approach, which enables the identification of the point and cause of bottlenecks in a practical and in-depth manner. This research design integrates quantitative and qualitative data, synthesizing the findings to provide a more comprehensive understanding of the issue.^19,20^ The aforementioned data collection methods were executed in parallel to gain a holistic and multidimensional understanding of the gap in the logic of the project and community context of service utilization. The design allows for a direct comparison of participants’ views derived from open-ended questions with perspectives derived from close-ended questions, effectively capturing both participants’ voices and statistical trends.^20^ In light of these merits, this methodology was deemed suitable for this study, given that the process evaluation of the MNH project necessitated field visits and surveys within a constrained timeframe to ascertain performance indicators within the social, cultural, economic, and health context of Tanzania.

**Figure 2:**
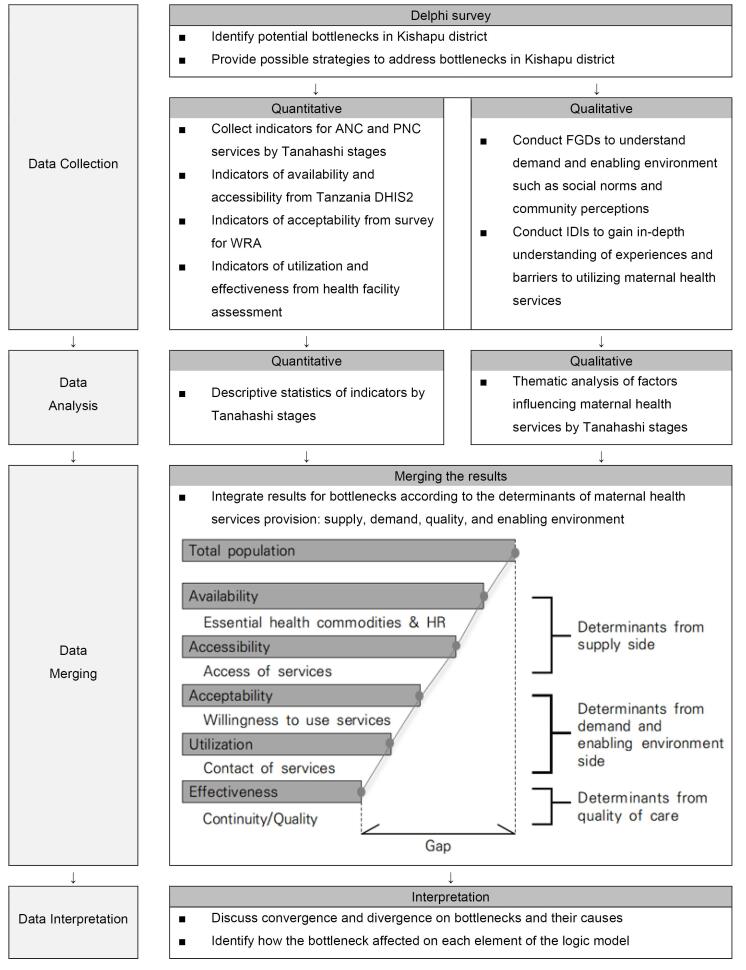
Study Overview With Convergent Mixed-Method Study Design and Tanahashi Framework. Abbreviations: DHIS2, District Health Information System 2; WRA, women in reproductive age; PNC, postnatal care; ANC, antenatal care; IDIs, In-depth interviews; FGDs, focus group discussions; HR, human resources.

 A Delphi survey with three occasions was conducted among local project executing agency officials and health experts to generate potential bottlenecks of MH interventions in developing countries. The health experts’ opinions were gathered to gain insight into contextual factors affecting utilization of services and to provide possible strategies to overcome bottlenecks in Kishapu district. Subsequently, a quantitative assessment was carried out to identify which domain of the Tanahashi Framework was experiencing the greatest challenges. In the next phase, the bottlenecks and potential alternatives were explored through a qualitative approach (Figure 2).

###  Participants and Data Collection

 In this study, project stakeholders were selected as qualitative research participants, including women in reproductive age (WRA), pregnant women, husbands, mothers-in-law, CHWs, and HP. These groups were chosen for their potential to provide generalizable insights into the research topic.^22,23^ Quantitative data were obtained from three sources: health facility assessment data, survey data for WRA (Supplementary file 1), and District Health Information System 2 (DHIS2), which is Tanzania’s national health statistics system.

 The health facility data was used to indicators on the availability and accessibility of ANC and postnatal care (PNC) services. Health facility data was collected from 49 facilities in Kishapu district (two facilities in Sekebugoro and three facilities in Itilima). The survey sampled WRA in the target area and collected data of the acceptability of ANC and PNC services. The sample size was calculated using the number of WRA and households in Kishapu, the effect size, and the confidence level (Supplementary file 2). The survey was conducted among 400 WRA living in Kishapu district (24 women in Sekebugoro and 21 women in Itilima). The DHIS2 contains information on the utilization of healthcare services by all pregnant women in the region, which can be used to collect data of utilization and effectiveness. Data from the Tanzania DHIS2 included 17 239 pregnant women in Kishapu region (1061 women in Sekebugoro and 1202 women in Itilima).

 Qualitative interviews were conducted by a team of two local consultants with the use of interview guidelines. A focus group discussion (FGD) was conducted for each group, comprising WRA, their husbands, mothers-in-law, CHWs, and HP. As previously stated, cultural norms and community perceptions represent significant determinants of healthcare utilization in Tanzania. Consequently, FGDs were conducted with members of the community to ascertain how prevailing perceptions of MH services influence their use from a demand-side perspective. Furthermore, FGDs were conducted with community members to determine how social norms are formed within this community as an enabling environment. It was anticipated that HP and CHWs would offer insights pertaining to the availability, accessibility and utilization of MH services from a supply-side perspective. WRA who are direct beneficiaries of the project were interviewed to gain an in-depth understanding of their experiences and barriers to utilizing MH services.

 The WRA were selected from among women who had given birth within the last three years and FGD and in-depth interview (IDI) were conducted separately for ANC and PNC. The research participants were divided on the basis of whether or not they had received ANC and PNC services. FGDs were conducted with 7 WRA who had used ANC, 14 WRAs who had used PNC, 10 husbands, ten mothers-in-law, 10 CHWs, and 5 HPs. Each target group was interviewed twice. IDIs were conducted with 10 WRA who had used ANC and ten WRA who had used PNC. The combination of these different approaches to data collection enabled the triangulation of data (Supplementary file 3).

###  Selection of Outcome Indicators

 The key outcome indicators are those identified in previous studies and in the expert Delphi as having a causal relationship with maternal mortality. From these, indicators were selected that are aligned with project activities and can be achieved in a relatively short period of time. In accordance with the Tanahashi model, this study employs three outcome indicators as variables. These correspond to the effectiveness stage indicators: the proportion of pregnant women who received initial ANC before 12 weeks at the last delivery, the proportion of pregnant women who received ANC at least four times at the last delivery, and the proportion of pregnant women who received PNC within 48 hours at the last delivery (Table).

**Table:** Operational Definition of Quantitative Indicators by Tanahashi Steps

**Indicators**	**Operational Definition**
**ANC**	
Availability of ANC	Proportion of health facilities that are available for ANC services Numerator: Number of facilities that can provide at least one of 7 ANC services Denominator: Total number of health facilities in Kishapu district Source: Health facility assessment
Accessibility of ANC	Proportion of wards (administrative region) where health facilities provide ANC services Numerator: Number of wards where health facilities provide ANC services Denominator: Total number of wards in Kishapu district Source: Health facility assessment
Acceptability of ANC	Proportion of WRA who recognize the need for ANC Numerator: Number of WRA who are aware that they need to receive initial ANC within 12 weeks of pregnancy Denominator: Total number of WRA who surveyed Source: Cross-sectional survey for WRA
	Proportion of WRA who are willing to receive ANC Numerator: Number of WRA who are willing to visit a health facility for ANC Denominator: Total number of WRA who surveyed Source: Cross-sectional Survey for WRA
Utilization of ANC	Proportion of pregnant women who received ANC at least once Numerator: Number of pregnant women who received ANC at least once during the most recent birth Denominator: Total number of pregnant women Source: Tanzania DHIS2
Effectiveness of ANC	Proportion of pregnant women who received initial ANC before 12 weeks Numerator: Number of pregnant women who received initial ANC before 12 weeks during the most recent birth Denominator: Total number of pregnant women in Kishapu district Source: Tanzania DHIS2
	Proportion of pregnant women who received ANC at least 4 times Numerator: Number of pregnant women who received ANC at least 4 times during the most recent birth Denominator: Total number of pregnant women in Kishapu district Source: Tanzania DHIS2
**PNC**	
Availability of PNC	Proportion of health facilities that are available for PNC services Numerator: Number of facilities that are available for at least one of delivery and basic emergency obstetric services Denominator: Total number of health facilities in Kishapu district Source: Health facility assessment
Accessibility of PNC	Proportion of wards (administrative region) where health facilities provider PNC services Numerator: Number of wards where health facilities provide PNC services Denominator: Total number of wards in Kishapu district Source: Health facility assessment
Acceptability of PNC	Proportion of WRA who are willing to receive PNC Numerator: Number of WRA who are willing to give birth at a health facility Denominator: Total number of WRA who surveyed Source: Cross-sectional Survey for WRA
Utilization of PNC	Proportion of pregnant women who delivered in a health facility at the most recent birth Numerator: Number of deliveries in a health facility Denominator: Total number of pregnant women in Kishapu district Source: Tanzania DHIS2
Effectiveness of PNC	Proportion of pregnant women who received PNC within 48 hours after the delivery Numerator: Number of pregnant women who received PNC within 48 hours after the delivery in a health facility Denominator: Total number of pregnant women in Kishapu district Source: Tanzania DHIS2

Abbreviations: DHIS2, District Health Information System 2; WRA, women in reproductive age; PNC, postnatal care; ANC, antenatal care.

 This study focuses exclusively on ANC services provided at health facilities, excluding outreach services from its assessment scope. PNC is also confined to the services provided within the context of health facilities. In the case of ANC, the results of the WHO randomized control trial and systematic review^24,25^ indicate that at least four ANC visits are required to improve MH. Moreover, the WHO 4-ANC model stipulates that the initial ANC visit should occur within the first eight to twelve weeks of gestation.^26^

 The qualitative items were developed on the basis of the results of the quantitative analysis, with the questions for each stakeholder organized around the items that emerged as bottlenecks in the latter. Questionnaires were constructed for WRA who are direct beneficiaries of the project to identify specific contexts affecting the utilization of MH services at each Tanahashi stage. Given the influence of husbands and mothers-in-law on WRA decision-making regarding the use of health facilities, the questionnaires were designed to capture a range of factor, including physical, economic, and cultural accessibility, as well as acceptability, encompassing cultural practices and family support. In order to ascertain the views of CHWs and HP, items were developed with a particular focus on the availability of MH services in health facilities, with specific attention paid to the levels of both availability and effectiveness (Supplementary files 4 and 5).

 Two regions were selected for the qualitative research through the local implementing agency, which allowed for a profound comprehension of the barriers to performance in the two regions. One region was selected where all three of the aforementioned effectiveness outcome indicators exhibited a notable increase from the baseline, surpassing the midline average. The other was chosen where two or more effectiveness outcomes fell below the average.

###  Data Analysis

 The objective of this study was to evaluate the progress of the MH project by analyzing the level of achievement of key performance indicators and the factors hindering performance. The principal outcome indicators pertained to ANC and PNC services. Furthermore, the regions with high (Sekebugoro) and low (Itilima) performance each were selected for comparison and analysis of the performance and bottlenecks in the two regions. A quantitative analysis was conducted using the Tanahashi model, with indicators applied at each stage as defined in the indicator matrix. This entailed a descriptive analysis.

 A team of two Tanzanian researchers conducted a qualitative analysis through thematic analysis.^19,20^ Thematic analysis was employed in order to identify the anticipated challenges that may arise in five specific stages delineated by Tanahashi. All semi-structured interviews were being recorded, transcribed and translated into English from Swahili, with a selection back-translated for quality assurance purposes. A matrix was devised for the collation of group information per question and thematic area. The responses were classified according to the question posed and the thematic area under consideration. The transcripts were coded according to an initial code list. To ensure inter-coder reliability, a minimal set of interviews was coded by two researchers, with one researcher checking the codes assigned by the other. In the event of any discrepancies, these were duly addressed and resolved through discussion. Once intercoder reliability had been established, a master code list was generated. Subsequently, the extracted themes and sub-themes were clustered in order to identify the underlying concepts. The sub-themes were organized by barriers regarding MH services, and the themes were organized according to the Tanahashi stage. The quantitative analysis was conducted using SPSS version 21, while the qualitative analysis was conducted using NVivo version 7.

## Results

###  Bottlenecks of Quantitative Research

 According to the quantitative results, service availability and physical accessibility were 100.0% and 98.0%, respectively, when using ANC services. There was no evidence of bottlenecks from the availability stage to the acceptability stage. However, despite the high numbers in these three stages, the utilization stage showed a low number of 17.4%, so it was decided that the first bottleneck had occurred. These results implied that barriers could arise from indicators not measured in the previous stages, such as the availability of healthcare services, economic accessibility, and intention to use services.

**Figure 3:**
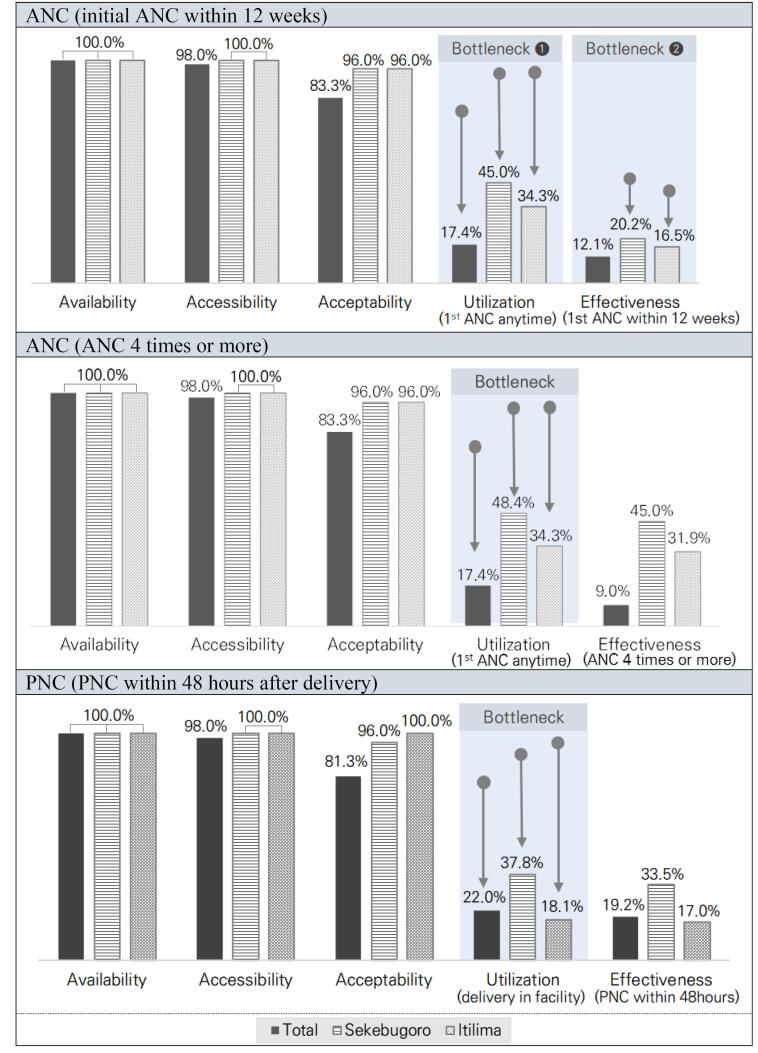
Bottlenecks of Antenatal and Postnatal Care by Quantitative Analysis. Abbreviations: PNC, postnatal care; ANC, antenatal care. Note: Sekebugoro and Itilima are the two areas in Kishapu district where interviews were conducted.

 Meanwhile, the indicators for PNC performed well compared to ANC. Similar to the ANC results, the previous three levels for PNC services had high numbers. However, the facility delivery rate, which is an essential prerequisite for the use of PNC as the utilization indicator, was 22.0%, indicating a bottleneck. Conversely, PNC within 48 hours after delivery was 19.2%, which was similar to the previous stage, confirming that the majority of facility deliveries result in PNC. As a result of the quantitative analysis, it showed similar patterns to the bottleneck location across the region. In other words, bottlenecks occurred at the ANC and PNC utilization stages. When looking at all regions, the bottleneck at the effectiveness stage was not noticeable due to the relatively low ANC utilization rate. However, compared to the utilization stage (Sekebugoro 48.4%; Itilima 34.3%), the rate of first ANC service use within 12 weeks (Sekebugoro 20.2%; Itilima 16.5%) decreased by more than half in both regions, so it was decided that a bottleneck had occurred. Although there was a difference in the absolute performance of the two regions, there was no significant difference in the Tanahashi stepwise analysis (Figure 3).

###  Bottlenecks of Qualitative Research

 Thematic analysis was conducted to identify barriers that may affect access to MH services at each level of Tanahashi. It also confirmed that barriers occurred at points that were not measured by quantitative data, as the barriers identified at each stage had a cumulative impact on the utilization and effectiveness stages (Supplementary file 6).

 At the availability stage, while there was no shortage of drugs or medical equipment, the lack of human resources appeared to be a serious problem. According to the Tanzanian government guidelines, health facilities should have a minimum of 15 staff at the dispensary level.^27^ However, most health facilities in the project area had 3 or 4 staff members, which was a significant barrier to service delivery. This limited staff was focused on urgent or emergency services, such as deliveries and surgeries. There is a need for more staff to address the needs of outpatients and assist them in accessing and providing essential MH services. In addition, HP were unable to check on pregnant women every six hours, which hindered the provision of PNC services.

 “*We are few and so sometimes they come here we are not around or they stay for too long so that demoralizes them, so even last year we were having low numbers of ANC attendances and children for immunization because of shortage of staff. We are only four of us” *(HP, FGD, region-Ilebelebe).

 In terms of accessibility, the health facilities in the project’s target region are public health facilities, so there should be no fees for ANC, PNC, or delivery services. However, some health facilities charged mothers for supplies that were difficult to keep in stock for delivery and PNC services, creating a financial burden for women.

 “*We don’t have enough water reserve, we have two tanks each 2000 liters which we use to harvest water during rainy season but that water cannot suffice the need throughout the year, so during dry season like this we usually use CHW to help us fetch water or sometimes relatives of women who come for delivery buy water at a cost of 300 per 20 liters” *(HP, FGD, region-Itilima).

 On the other hand, due to the lack of good roads and transport, the physical accessibility of a health facility was limited during the rainy season. It is a barrier to continued use of services. In Sekebugoro, health facilities were located along the paved road, but in Itilima, health facilities tended to be located more on the outskirts and were more affected by climatic factors.

 “*It is very far because I use 9 hours walking on foot, which means I cannot come here on foot. I have to use either bicycle or motorbike, there are no buses and during rainy season it is even worse, sometimes during rainy season it is difficult to get transport even when you have money” *(WRA, IDI-ANC 5, region-Itilima).

 The predominant factor contributing to this bottleneck was found to be sociocultural factors at the level of acceptability that negatively affect access to ANC services. Apart from women’s knowledge and perceptions, a social consensus on the need for ANC services has yet to be established in the target region of this project. A spouse or mother-in-law, who is the primary decision-maker for the family, also tended to have a negative perception of MH services due to a lack of knowledge. Interviews with CHWs and HP revealed that the knowledge and supportive behavior of spouses and family members were the most important factors in a pregnant woman’s decision to seek MH services.

 “*Low education on the importance of ANC visits is mainly found in families who are very remotely situated or those where the mother-in-law stays with his son because the mother-in-law significantly influences the pregnancy issues*” (CHWs, FGD, region-Sekebugoro).

 Similar issues were identified with PNC. The WRA had a perception that PNC was neonatal care, such as immunization, and the difference between delivery and PNC was not clearly recognized. As a result, it was common for women to be discharged within 24 hours of delivery, and there were many cases of women not receiving PNC despite staying at the facility.

 In the underperforming area of Itilima, traditional norms were found to be a strong barrier to accessing services. In the target area, pregnant women are expected to dress in a certain way when they leave the house. If she is not dressed appropriately, she may receive inappropriate comments from strangers and health facility staff. In addition, using the health facilities is considered as a “special outing” for women; therefore, it is customary for them to wear colorful new clothes when they seek services, although the same is not true for men. Thus, customs and norms have created a burden that limits women’s use of services at health facilities.

 “*I did not come on time on my first visit because my husband did not buy me clothes for pregnancy (loose gown- Dera in local language), nurses usually are very furious when they see me with clothes that tighten our skin, so I had to wait until he got money to buy me that special dress” *(WRA, IDI-ANC 5, region-Itilima).

 Previously, it has been shown that the normative barriers to utilization also affect the effectiveness level. Inadequate support from family members and underestimation of the effectiveness of ANC services created a burden for women to continue using services. Thus, it served as a barrier not only to the utilization stage of initial ANC use, but also to continued use of services. The expectation that women should stay at home and do household chores rather than seek ANC early, also appears to have acted as a barrier to seeking the first ANC within 12 weeks.

 “*My husband did not accept the pregnancy, so I decided not to take care of it, and so not to attend ANC because they said I had to come with my husband and it was a must” *(WRA, IDI-ANC 4, region-Sekebugoro).

 Even when women were aware of ANC, they could not take their first ANC in a timely manner because they did not know if they were pregnant themselves. In addition, women did not continue to visit the health facility for ANC services because of a negative initial experience. In order to provide standardized ANC services in all health facilities, capacity building training had been implemented for HPs in the target area of the project. However, there was still a large gap in the ability of staff to provide services, which meant that pregnant women did not have access to a consistent range of services. The low quality of services at the health facilities was due to the accumulation of negative experiences by the women who had sought services, resulting in the facilities having a negative reputation among the mothers’ peer groups and the community and acting as a bottleneck to service utilization.

 “*I know I should come at 12 weeks, but I came late at 16 weeks because I had abdominal pains at home so I was treating my abdominal pains locally. Furthermore, I became conscious of pregnancy at 16 weeks when I felt dizziness, General body weakness then I came to dispensary for diagnosis. To me missing periods is not a reliable sign of pregnancy as I can miss my periods several months while I am not pregnant” *(IDI, WRA, region-Sekebugoro).

###  Integrated Results for Bottlenecks of Providing Maternal Health Services 

**Figure 4:**
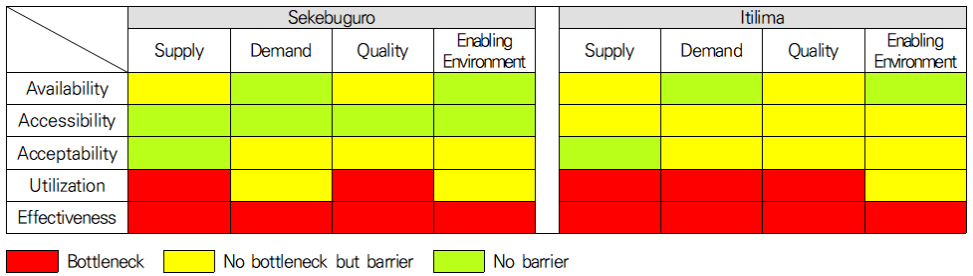
Barriers of Providing Maternal Health Services by Regions.

 Quantitative and qualitative analyses showed that the capacity of health facility staff, the level of service delivery, the accessibility of health facilities, and both knowledge and awareness of the use of MH services in the community have improved. However, the qualitative analysis confirmed that barriers still exist at all stages of service use. Although there were no problems in achieving the quantitative indicators of each stage, it seems that this did not lead to achieving the indicators of the next stage. This means that a bottleneck occurred at a particular stage due to the accumulation of problems from previous stages. In addition, if quantitative results show that the inputs and activities in the logic model were achieved but did not lead to the use of outputs, it means that something was not considered when constructing the logic model. Barriers found in qualitative results can be viewed as missing premises in the logic model. The barriers to the provision of MH services were suggested by the determinants of health service provision: supply, demand, quality, and the enabling environment.^8^ As a result of integrating the quantitative and qualitative findings, the barriers to the provision of MH services in two comparison regions are as follows (Figure 4).

 First, on the supply-side, the lack of human resources and the shortage of medicines were barriers. Staff shortage at health facilities resulted in long waiting times, leading to dissatisfaction and a negative perception of the facility and its services, which discourages expectant mothers from using the service both then and in the future. In addition, stock-outs sometimes required women to pay for drugs or a service fee, creating unanticipated economic constraints. While the national guidelines require a minimum of 15 staff to run a dispensary, most facilities have only 3 or 4 staff.^28^ Despite the support of CHWs, staff were mainly used for urgent deliveries or emergency services, and it seemed difficult to smoothly provide staff for ANC and screening, which are relatively low-risk.

 Roads and transportation were another supply-side barriers that limit facility access and using ANC and PNC services. In the Kishapu area, if a family is in a remote location that is difficult to access, the CHWs would have difficulty visiting the family. The family also has difficulty getting to the health facility, especially during the rainy season when the roads disappear. This project provided an ambulance for both emergency and general use, and as an alternative, CHWs could transport expectant mothers by bicycle. However, these methods could only help a few eligible people, so more universal options need to be established.

 “*I failed to deliver at the health facility because my husband was not around, and there was no one to escort me to the health facility, also I had no money to take taxi or motorbike to the facility. It was during the rainy season which is always difficult to pass across the clay muddy road between our village and the dispensary” *(WRA, IDI-PNC 1, region-Itilima).

 Second, on the demand-side, inaccurate knowledge of MH services by primary family decision-makers could lead to inappropriate supportive behaviors and limit women’s access to health facilities. In an environment where a woman’s independent decision-making is limited, the economic and emotional support of her family members could become a significant barrier to the use of MH services. When it is the first pregnancy or when the mother is ill, they are active in using health facilities. But when the number of pregnancies increases or when the mother is healthy, there was a perception that it is not necessary to seek medical services at health facilities. In addition, in the early stages of pregnancy or just before delivery, family members showed supportive behaviors such as visiting health facilities together, but in mid-pregnancy, there were more cases of pregnant women visiting health facilities alone for continuous ANC.

 Third, as an enabling environment, a lack of knowledge about MH and social norms or practices were significant barriers to using health facility services. Demand-side barriers and the enabling environment were closely linked. According to the survey results, WRA in the target area recognized the need for ANC and facility delivery. They intended to use the health facilities, but there was a gap between intention and actual use of services. In particular, the lack of knowledge about MH was more pronounced for PNC than for ANC. Compared to ANC, the conceptualization of PNC needed clarification and was mistakenly understood as only neonatal care. Superstitions and customs about MH were widespread and hindered access to health facilities. Even if a pregnant woman had a positive attitude towards using a service at a health facility and wanted to use it, strong cultural taboos in the community could prevent her from accessing the facility.

 Finally, in terms of service quality, if a previous experience of using MH services was unsatisfactory or inadequate, it would affect one’s continued use of the service in the future. According to the quantitative analysis, the prevalence of receiving ANC first-time was 17.4% and the percentage of receiving ANC within 12 weeks was 12.1%. Although most women who received ANC at least once received it early, the proportion of women who received ANC at least four times before delivery was relatively low at 9%. Inconsistent services from facility to facility, unfriendly attitudes of HP, or gaps in the capacity of individual HP can lead to negative experiences. These negative experiences led to a decline in the continued use of MH services. Standard guidelines and regulations needed to be adequately implemented, and the level of quality needed to be more consistent across facilities. Rather than providing a standardized care, HP appeared to have arbitrarily decided what services to provide to individual case.

## Discussion

 This study identified significant barriers to the MH project related to ANC and PNC services. For both, barriers were found in the actual use of services. For ANC, the rate of continuous use more than four times (9.0%) or timely use within 12 weeks (12.1%) was low compared to the rate of first-time use of ANC at any time (17.4%). Previous studies^2,8,29^ also found low effective coverage of ANC care. On the other hand, for PNC, the rate of facility delivery was slightly lower (22.0%), but the continuous PNC utilization within 48 hours after delivery was 19.2%. This trend was evident in both regions.

 Given that the bottleneck was not identified until the acceptance phase, community awareness and acceptance appeared to be high, but only half of them had received services. In other words, although interventions to improve knowledge and awareness were implemented according to the logic model, these activities were not sufficient to change health behaviors. This means that even when infrastructure such as equipment and facilities are in place, people may not use the service due to contextual factors such as norms and perceptions. Also, even if they do use the service, they may not use it in a timely and consistent manner due to low service quality or unsatisfactory experiences. The logic model for this project seemed to have been designed to focus on the infrastructure and accessibility aspects of services, and it should be noted that cognitive and cultural factors play a complex role in the decision to use services.

 Previous studies from Tanzania found that cultural norms, socioeconomic factors, and traditional beliefs shaped a pregnant woman’s access to health services and her choice of delivery location, with these decisions frequently being made by her mother-in-law or husband rather than by the woman herself.^6,27,30^ Grandchildren are considered part of the paternal lineage, which places a responsibility on mothers-in-law to care for them. Additionally, pregnant women are compelled to adhere to the decisions of their mothers-in-law, who possess greater decision-making power, in order to secure emotional and social support.^31^ Furthermore, in Tanzania, husbands maintain control over resources and financial decisions, which often results in women relying on their husbands to cover the costs associated with visiting health facilities.^32^

 In line with previous research findings, interviews in this study revealed that WRA perceived their spouses or mothers-in-law as decision-makers, and their lack of knowledge or negative perceptions of MH services made it difficult for them to visit a clinic. CHWs and HP also agreed with that the knowledge and supportive behavior of spouses and family members were the most important factors in a pregnant woman’s decision to seek MH services. In Sekebugoro, it appears that nuanced awareness-raising activities, such as photovoice, which were not conducted in other regions, led to higher actual medical utilization. In Itilima, not only did traditional norms have a stronger effect than in other regions, but physical accessibility was also low, so there were high barriers to visiting health facilities and using services.

 On the other hand, based on interviews with HP and CHWs, they emphasized that there were still physical bottlenecks on the supply-side. In many developing countries, MH programs also faced barriers at various stages. In particular, barriers to availability and accessibility, the supply-side of service delivery, were prominent.^5,8,33-35^ Providing quality healthcare was challenging because they had to provide healthcare with limited resources. In this study, the qualitative research uncovered barriers that were not identified by quantitative analysis and confirmed the “cumulative” effect of problems at all stages. Previous studies using the Tanahashi method have interpreted the problems at each stage,^2,35^ but this study identified how the barriers at each stage are related and interact. In addition, this study identified barriers from multiple perspectives by listening to key stakeholders on both the supply- and demand-sides of MH service utilization.

 This study found that the barriers caused by the missing assumptions in the logic model affected the achievement of project outcomes. It is necessary to properly establish a causal relationship as to whether the performance achieved in the output can logically contribute to the achievement of the results. It is also necessary to establish a system of indicators that provide a logical link. This requires a sufficient discussion with various stakeholders from the beginning of the project, including the identification and definition of performance indicators and the collection of specific data.

 To overcome the above barriers, the logical link between input-activity, activity-output, and output-outcome should be strengthened. First, in terms of the input-activity linkages, gaps in workforce capacity not only act as a barrier during the availability phase of service delivery, but also have a significant impact on service quality.^5,8,36^ Quality management involves ensuring that all health workers are provided with adequate training opportunities. CHWs are the “bridge” between beneficiaries and service providers. As they are the closest personnel to the community, they need to be professionalized through regular training, including remuneration and patient management. In the long term, CHWs need to be integrated and managed into the health system. Furthermore, at the national level, consideration should be given to building an overall infrastructure through the supply and recruitment of health workers and the ongoing capacity building to develop a skilled health workforce.

 In addition, barriers of accessibility and acceptability arose when activities did not result in outputs. It is necessary to change norms through influential figures in the community, such as opinion leaders, and to implement interventions that lead to specific supportive behaviors among key stakeholders, not just to change their perceptions. Awareness-raising and knowledge-building activities that can lead to health behavior change, such as photovoice and community seminars, should be delivered repeatedly and consistently to community members. Information on how to visit health facilities should be provided to community members through CHWs, and arrangements should be made for transportation using various modes of transport.^8,35^

 Next, the issues to be considered in linking outputs and outcomes should mediate the influence of contextual factors. Qualitative findings confirmed that timely and continuous access to health services depends on the beneficiaries’ experience and satisfaction with the services they received. Therefore, it is necessary to improve not only the professionalism of health workers through education and training, but also the quality of services and providers’ attitudes toward patients. It is also necessary to find ways to encourage continuous visits and to establish a feedback system for negative experiences in health facilities.^35,37^

 Strengthening ownership and community awareness can help build a sustainable service delivery system. As stated in the Organisation for Economic Co-operation and Development’s Development Assistance Committee evaluation criteria,^38^ strengthening the sense of ownership of recipient countries and beneficiaries is an important factor in sustaining project performance.^39^ To this end, it is necessary to increase the applicability and concreteness of alternatives that can contribute to improving MH by actively sharing process evaluation results with the community.^8,40^ In addition, it is necessary to institutionalize regular sharing of results and active collection of opinions on project performance and barriers with implementing agencies, HPs, and CHWs working in the project area.^39^

 The process evaluation of the Tanzania MH project analyzed barriers to achieving outcomes and proposed alternatives to complement the logic model. The barriers identified in this study should be considered in the planning of future MH projects. For projects already underway, project priorities can be adjusted to focus on identified bottlenecks in different regions. However, as shown above, monitoring and adjustment throughout the service delivery process is still important, as problems from the earlier stages can accumulate and affect later stages.

 In this study, outcome indicators were selected based on the project’s integrated indicators. Therefore, it was difficult to measure performance or barriers that occurred at points not included in the performance indicators. As a result, there were limitations in disaggregating indicators to each stage and in clearly distinguishing obstacles by bottleneck stage or region. In follow-up research, it is suggested that the indicators be disaggregated and measured at each stage based on the barriers identified in this study. It is also necessary to include MH outcomes as indicators to evaluate the performance of the project. This study only includes a cross-sectional evaluation at the midpoint of the program, and subsequent studies should evaluate outcomes and barriers throughout the project period.

 In addition, because the barriers were measured in the project implementation and target areas, barriers that occurred at the macro level could not be addressed. Therefore, there are limitations in generalizing them as barriers in other communities. A health system, organization, and leadership are critical latent variables in all stages of Tanahashi. To promote MH, it is necessary to interpret them considering the characteristics and context of the community.

## Conclusions

 Contextual factors not considered for in the logic model of MH programs can act as cumulative barriers, that affect health outcomes. Improving provider attitudes, service quality, and the supportive climate of family and community members is necessary to ensure that target populations are satisfied with and continue to use services. To improve coverage of MH services, ongoing activities to change stakeholders’ perceptions of MH and service utilization behaviors should be included. Strengthening the MH infrastructure, supported by policy and government actions, is also essential to reduce maternal mortality in the long term.

## Acknowledgements

 We would like to express our gratitude to Korea International Cooperation Agency (KOICA) for supporting the implementation of this evaluation of the MNH project in Tanzania. Also, we are thankful for Good Neighbors for their cooperation in carrying out this study in the project area and share project’s performance data.

## Ethical issues

 Approval for the process evaluation of the Tanzania Maternal and Child Health Program was obtained from the Ewha Womans University Institutional Review Board on August 1, 2017 (Ewha Womans University Bioethics Committee No.139-9). The Tanzania field study was approved by the National Institute for Medica Research Dar Es Salaam on July 31, 2017 (NIMR/HQ/R.8a/Vol.2017).

## Conflicts of interest

 Authors declare that they have no conflicts of interest.

## Supplementary files


Supplementary file 1. Survey Questionnaire for Women of Reproductive Age.

Supplementary file 2. Calculate Sample Size for Survey of Women in Reproductive Age.

Supplementary file 3. General Characteristics of Quantitative and Qualitative Study Participants.

Supplementary file 4. Focus Group Discussion Guide.

Supplementary file 5. In-Depth Interview Guide.

Supplementary file 6. Theme-Code Structure of Thematic Analysis.
