# Supplementary material for: Bottlenecks Analysis in the Intervention of Improving Maternal Health in Rural Areas of Tanzania: A Convergent Mixed-Method Approach
Source: Int J Health Policy Manag. 2025 Mar 2;14:8355. doi: 10.34172/ijhpm.8355 (PMC12032231; doi:10.34172/ijhpm.8355)
Supplement: Supplementary file 1 — Survey Questionnaire for Women of Reproductive Age. [file ijhpm-14-8355-s001.pdf]

Article title: Bottlenecks Analysis in the Intervention of Improving Maternal Health in Rural Areas of Tanzania: A Convergent Mixed-Method Approach

Journal name: International Journal of Health Policy and Management (IJHPM)

Authors' information: Hyeyun Kim[1,2], Jiye Kim[2], Seohyeon Lee[2], Minkang Cho[2], Hyekyeong Kim[3]*

[1]Korea Institute for Health and Social Affairs, Sejong, Republic of Korea.

[2]Department of Health Convergence, Graduate School of Ewha Womans University, Seoul, Republic of Korea.

[3]Department of Health Convergence, Ewha Womans University, Seoul, Republic of Korea.

***Correspondence to:** Hyekyeong Kim; Email: hkkim@ewha.ac.kr

Citation: Kim H, Kim J, Lee S, Cho M, Kim H. Bottlenecks analysis in the intervention of improving maternal health in rural areas of Tanzania: a convergent mixed-method approach. Int J Health Policy Manag. 2025;14:8355.doi:10.34172/ijhpm.8355

Supplementary file 1. Survey Questionnaire for Women of Reproductive Age

QUESTIONNAIRE FOR WOMEN OF REPRODUCTIVE AGE

Dododoso la akina mama walio katika umri wa kuzaa

KNOWLEDGE, AWARENESS AND UTILIZATION OF SKILLED BIRTH ATTENDANTS FOR RMNCH SERVICES AMONG WOMEN (15-49 YEARS) IN KISHAPU DISTRICT, SHINYANGA, TANZANIA.

**Nearest health facility:** *Jina la kituo cha afya kilicho jirani*<br><br>……………………………….<br><br>**Level of nearest HF:** *Ngazi ya kituo*<br><br>…………………………….	**Name of district:** *Wilaya*<br><br>……………………………<br><br>**Name of ward:** *Kata*<br><br>…………………………..<br><br>**Name of village/street:** *kijiji/mtaa*<br><br>……………………………<br><br>**Urban or rural:** *mjini/kijiji*<br><br>………………………………	**Date of interview:** *Tarehe ya mahojiano*<br><br>……………………………<br><br>**Name of interviewer:** *Jina la anayehoji*<br><br>……………………………<br><br>**Tel of interview:** *Namba ya simu ya anayehojiwa*<br><br>……………………………<br><br>**Date of Last Live Delivery:** *Tarehe ya kujifungua mtoto hai mara ya mwisho* **(dd/mm/yyyy)……………**

Ⅰ. Socio – Demographic Characteristics

1. Date of birth of woman in years *Tarehe ya kuzaliwa (DD MM YYYY)*……………………………

2. Age of the participant in years *Umri jaza miaka iliyokamilika*…………………………………….

3. Marital status *Hali yako ya ndoa kwa sasa ikoje?*

1) Married *Nimeolewa*
2) Cohabiting *Tunaishi bila ndoa*
3) Single *Sijawahi kuolewa*
4) Divorced/Separated *Talaka/Tumetengana*
5) Widow *Mjane*

4. (a) If Married/Cohabiting for how many years are you living together? ……………………………….
Kama umeolewa au kuishi bila ndoa, je mmeishi pamaoja na mwenzi kwa muda gani?

(b) Separated/Divorced/Widow for how many years? …………………………………………….
Kama ni mmetengana, mtalaka au mjane umekaa hivyo kwa muda gani?

5. Education level *Kiwango cha elimu cha mshiriki*

1) Never been in school *Hakusoma*
2) Primary Education *Elimu ya msingi*
3) Secondary Education (''O" or ''A" level) *Elimu ya sekondari*
4) Tertiary Education *Elimu ya juu*

6. Are you employed and you receive regular month salary?
Je umeajiriwa na unapokea mshahara kila mwisho wa mwezi?

0) No *Hapana* 1) Yes *Ndiyo*

7. Occupation *Kazi* 1) Civil Servant *Mtumishi wa serikali*

2) House wife *Mama wa nyumbani*

3) Peasant *Mkulima mdogo*

4) Small business *Biashara ndogondogo*

5) Other specify……………………………………………………………

8. Approximate income of the woman per month? *Kwa kukadiria kipato chako kwa mwezi ni kiasi gani?* Tanzanian shs ……………………………….*(If doesn't have record 0)* **[ACCESS]**

9. What is approximate family income per month (yours partner plus your income)? **[ACCESS]**
Kwa kukadiria je kipato cha familia ni kiasi gani ukiweka chako na cha mwenzi?

Tanzanian shs……………………………….

10. Who is a decision maker in your family? **[ACCEPT]**

1) Husband
2) Mother-in-law
3) Father-in-law
4) Other specify……………………………………………………..............

11a. What is the nearest health facility **with antenatal care services? [ACCESS]**
Kliniki ya karibu inayotoa huduma za mama na mtoto?

Name (of health facility)……………………………………………

Is it a 1) Dispensary *Dispensari/zahanati*

2) Health centre *Kituo cha afya*

3) Hospital? *Hospitali*

11b. Can you mention all the reproductive, maternal, newborn and child health services which you are aware a woman can get at nearest health facility mentioned in Q 11a? **[ACCESS, ACCEPT]**
Je ni huduma zipi za uzazi, mama na watoto zilizo katika kliniki iliyo karibu na wewe?

11c. Of the mentioned reproductive, maternal, newborn and child health services in Q 11b, which ones have you used in the past 5 years? **[UTILIZE]**
Je katika huduma ulizotaja swali 16b, ni huduma zipi za uzazi, mama na watoto zilizo katika kliniki iliyo karibu na wewe ulizotumia au kupata katika kipindi cha miaka 5 iliyopita?
……
……
………………………………………………………………

12a. What is the nearest health facility with **delivery services**? **[ACCESS]**
Kliniki ya karibu inayotoa huduma za kujifungua?

Name……………………………………

Is it a 1) Dispensary *Dispensari/zahanati*

2) Health centre *Kituo cha afya*

3) Hospital? *Hospitali*

12b. What is the distance from home to the nearest health facility with delivery services when using a car or motorcycle? *Ukitumia pikipiki au gari, je kuna umbali gani toka kwako hadi ufike kituo cha karibu chenye huduma za kuzalisha?* **[ACCESS]**

1) Up to 30 minutes *hadi dakika 30*

2) 31 minutes to 60 minutes *dakika 31 hadi 60*

3) 61 – 120 minutes (> 1 hour to 2 hours) *dakika 61 hadi 120*

4) 121 minutes – 180 minutes (> 2 hours to 3 hours) *dakika 121 hadi 180*

5) > 181 minutes (More than 3 hours) *zaidi ya dakika 180*

12c. What is the fare you use to pay from home to the nearest health facility with delivery services when using a car or motorcycle? *Je unalipa kiasi gani ukitumia pikipiki au gari, ili ufike kwenye kituo cha afya cha karibu chenye huduma za kuzalisha?* **[ACCESS]**

1) Fare by using motor cycle in TZS *Nauli kwa pikipiki*………………….…………………………

2) Fare by using public transport (hiace) in TZS *Nauli kwa kibasi*…………………………….

3) Fare by using hired car in TZS *Nauli kwa taxi au gari la kukodi*…………………………….

12d. What is **the common mode of transport** doing most women use when they go for delivery at your area? *Kwa kawaida usafiri gani unatumiwa na wamawake walio wengi wanapokwenda kujifungua?* ………………………………………………….………………………………………………… **[ACCESS]**

II. Reproductive & Maternal health history

13a. Number of pregnancies *Umeshawahi kuwa mjamzito mara ngapi?*…………………………………

13b. Total number of **births** in the past 5 years ……………………………………
Jumla ya watoto aliojifungua kwa miaka mitano iliyopita

13c. Total number of **live births** in the past 5 years …………………………………

Jumla ya watoto walio hai aliojifungua kwa miaka mitano iliyopita

Antenatal care for last pregnancy

14. Do you know when is the proper timing for the first antenatal care? **[ACCESS]** **[Outcome 3d]**

1) Within 12 Weeks 2) within 16 weeks 3) within 20 weeks 4) within 24 weeks 5) don't know

15. In general when/ at what gestation age do the women start to attend for antenatal care in this area?

Kwa kawaida katika eneo hili, wanawake wanaanza kuhudhuria kliniki mimba ikiwa na umri gani?.. **[ACCEPT]**

16. Do you know how many times pregnant women should have ANC(antenatal care)? **[ACCESS]**

1) 1 time 2) 2 times 3) 3 times 4) 4 times 5) 5 times 6) Every month

17. Did you attend/go for antenatal care/ clinic during your **last pregnancy? [UTILIZE]**
Ulihudhuria kliniki kwenye ujauzito wako wa mwhisho?

0) No 1) Yes

18. Which level of care did you get your antenatal care for the last pregnancy? **[UTILIZE]**
Je ni kituo kipi ambacho ulipata huduma za afya katika ujauzito wa mwisho?

1) Dispensary *Dispensari/zahanati* (Name *Jina*.....................................)

2) Health Center *Kituo cha afya* (Name *Jina*..)

3) Hospital *Hospitali* (Name *Jina*..)

19. What was the gestation age on the first visit? *Mimba ilikuwa na umri gani* ***kwa miezi*** *ulipohudhuria kliniki kwa mara ya kwanza?* ....**[UTILIZE]**, **[Outcome 2b]**

20. How many times did you attend for antenatal care? *Ulihudhuria kliniki mara ngapi hadi ulipojifungua?* .... **[EFFECT], [Outcome 2a]**

ANC 1 __________Weeks (Name of place:________________________)
ANC 2 __________Weeks(Name of place:________________________)
ANC 3 __________Weeks(Name of place:________________________)
ANC 4 __________Weeks(Name of place:________________________)

21a. Were you satisfied with antenatal care you received during your last pregnancy? **[EFFECT]**
Je, uliridhishwa na huduma uliyopewa katika kituo cha afya kwenye ujauzito wako wa mwisho?

0) No *Hapana (sababu za kutoridishwa?..)*

1) Yes *Ndiyo*

21b. If you were not satisfied, what were the reasons?(Can choose multiple answers)

1) The facility did not have enough drugs, beds, rooms etc.
2) The facility was not clean
3) Unkind attitudes of health workers

4) Skilled healthcare workers were not present (or not enough) in the facility
5) Long time to get the service
6) Difficult to understand of explanation
7) High cost
8) Other______________

22. If you did not have pregnancy checkup at least four times, why? **[EFFECT]**

1) I believed that I had already enough check-up(s)
2) It felt like a burden to visit a health facility several times because it was too far from my home
3) Financial concerns
4) No transportation, or transportation was not available
5) Other (specify)___________________

23a. Did you get any information on the pregnancy check-ups or antenatal care from CHW? **[ACCESS]**

0) No 1) Yes

23b. If yes, what was about it? (Check below) **[ACCESS]**

1) Regular check-ups and the proper timing for check-ups
2) Related MNCH services of health facilities
3) Danger signs of childbirth
4) Delivery complications
5) Obstetric emergencies and referral
6) Others: ___________

24. Who was attending you most of the times when you attended for ANC? **[ACCESS]**
Nani alikuwa anakupa huduma mara kwa mara katika ujauzito wako wa mwisho?

Ulihudhuria kliniki kwenye ujauzito wako wa mwhisho?

1) Nurse/ midwife
2) Doctor
3) Other please mention……………………..…………………………..

25. Were the following measured or performed during your **last pregnancy**? **[UTILIZE]**
Je vipimo vifuatavyo vilifanyika wakatiwa ujauzito wako wa mwisho?

	Category	0. No	1. Yes	2. Don't know
A	Blood pressure measured *Kupimwa shinikizo la damu*			
B	Weight taken *Kupimwa uzito*			
C	Blood checked for HIV *Kupima virusi vya UKIMWI*			
D	Syphilis *screening Kupimwa kaswende*			
E	Hemoglobin *test Kupimwa wingi wa damu*			
F	Urine taken *Kupimwa mkojo*			
G	Given SP (IPT) to prevent malaria *Alipewa Sp (IPT) kuzuia malaria*			

If given IPT how many times?.Kama alipewa ni mara ngapi?... [**Outcome 2c]**

Place of delivery for last pregnancy *Alipojifungulia ujauzito wa mwisho*

26. Where did you give birth of your last baby? (*last born) Je mtoto wa mwisho ulijifungulia wapi?* **[UTILIZE]**

1) Health facility *Kituo cha afya*
2) Home *Nyumbani*
3) Other *Kwingine*…………………………………………………………

27. Which level of health facility did you deliver your last baby? **[UTILIZE]**
Jinan a ngazi ya kituo alichojifungulia mtoto wa mwisho

Name (of health facility)……………………………………………

1) Dispensary *Dispensari/zahanati* (Name *Jina*……………………………….)
2) Health Center *Kituo cha afya* (Name *Jina*…………………..………………)
3) Hospital *Hospitali* (Name *Jina*……………………………………………………..)
4) Not applicable because delivered at home *alijifungulia nyumbani*

28. Who assisted with delivery of your last child? *Nani alikuzalisha?* **[UTILIZE]**

1) Health Care provider *Mtaalamu wa afya*
2) Midwife
3) TBA *Mkunga wa jadi*
4) Relative/Mother/Mother in law *Ndugu/mama mzazi/mama mkwe*
5) Others mention *Wengine taja*……………………………………………….

29. After delivery of your last child, how long did you stay at the hospital before discharge? **[EFFECT]**
Je b*aada ya kujifungua mtoto wako wa mwisho, ulikaa hospitali au kituo cha afya kwa muda gani kabla ya*

0) Not applicable delivered at home

30a. Were you satisfied with care you received **during delivery** at the health facility? **[EFFECT]**
Je uliridhishwa na huduma ulizopewa katika kituo ulichojifungulia mtoto wako wa mwisho?

0) No 1) Yes 2) Not applicable because delivered at home **(Go to 30c)**

30b. If not, what were the reasons? (multiple responses possible) **[EFFECT]**

1) The facility (or the health worker) did not have enough drugs, beds, mattresses, rooms, etc

2) The facility was not clean
3) Skilled healthcare workers (doctor, nurse, midwife, or physician's assistant) were not present in the facility or there were not enough healthcare workers there
4) Long time to get to the facility (or to see the healthcare worker)
5) Long time to get the service
6) Health staff and skilled healthcare workers' explanations were difficult to understand
7) High cost
8) Other (Specify): ______________________

30c. Why <u>did you deliver your last baby</u> at home? *Ni kwanini uliamua kujifungulia nyumbani?* **[ACCESS]**

1) Transportation issues
2) Long travel time
3) Financial concerns
4) Health facility issues (including health workers' issues)
5) Other pregnant women in the community usually do NOT go to a health facility
6) Stay home alone/No one caregiving
7) Partner/mother-in-law don't allow to go to check-up
8) Other (Specify):

35a. Which care/services; were you given yourself? *Wewe binafsi ulipewa huduma gani?* **[UTILIZE]**
……
………………………………………………………………………………………………………

35b. Services given for the baby? *Mtoto alipewa huduma gani?* **[UTILIZE]**
………

…….

<u>Postnatal care for last pregnancy</u>

31. Did you go/attend for post-natal check up at the health facility after delivery of **your last born** baby?
Je, ulihudhuria kituo cha afya ili kupata huduma baada ya kujifungua mtoto wako wa mwisho?
[UTILIZE]

0) No 1) Yes

32. How long after delivery did you go for 1st check up? *Ulichukua muda gani baada ya kujifungua kwenda kupata huduma kliniki kwa mara ya kwanza?* ……………………………………days **[UTILIZE], [Outcome 2e]**

33. How frequent did you attend for post-natal care from delivery to 40 days post partum? *Je ni mara ngapi umehudhuria huduma za kliniki ndani ya siku 40 baada ya kujifungua?* **[EFFECT]**

1) Once/*Mara moja*

2) Twice/ *Mara mbili*

3) Three or more/ *Zaidi ya mara tatu*

34. Where did you attend for postnatal check-up? *Ulikwenda wapi kwa ajili ya ufuatiliaji wa afya yako?* **[UTILIZE]**

1) Dispensary *Dispensari/zahanati* (Name *Jina*......................................)
2) Health Center *Kituo cha afya* (Name *Jina*.......................................)
3) Hospital *Hospitali* (Name *Jina*...)

36. In general, what were you counseled about during postnatal care visits? **[UTILIZE]**
Je unapokuja kliniki baada ya kujifungua umeshapewa ushauri kuhusu mada zipi?.

....
....

37. Reasons for do-not-utilize maternal services in health facilities

37a. Why did not you go to anyone for the pregnancy check-ups(or antenatal care)? **[ACCESS]**

(Multiple responses possible, circle all mentioned responses)

1) I didn't know whether I was pregnant
2) I didn't know where to go
3) Transportation issues -> Go Q 37b
4) Long travel time
5) Financial concerns
6) Health facility issues (including health workers' issues) -> Go Q 37C
7) Other pregnant women in the community usually do NOT go to a health facility
8) Stay home alone/No one caregiving
9) Partner/mother-in-law don't allow to go to check-up
10) Other (Specify): ______________________
11)

37b. If you choose transportation issues, what are the reasons? **[ACCESS]**

(Multiple responses possible, circle all mentioned responses)

1) No transportation, or transportation was not easily accessible
2) The quality of transportation was bad
3) Other (specify)___________________________

37c. If you choose health facility issues, what are the reasons? **[ACCEPT]**

(Multiple responses possible, circle all mentioned responses)

1) I don't believe healthcare facilities are clean
2) I don't believe healthcare facilities have enough beds or rooms for delivery
3) I don't believe health workers (doctor, nurse, midwife, or physician's assistant) are present in the facility or NOT enough health workers are present
4) Other (Specify): ______________________

Ⅲ. Future use of health facilities *Matumizi ya vituo vya afya kwa mimba zijazo*

38. Are you planning to have more babies? *Je una mpango wa kupata watoto wengine?*

0) No 1) Yes

39. Will you attend for ANC at the facility in case you are blessed with another pregnancy? **[ACCEPT]** *Je utahudhuria kliniki iwapo utajaliwa ujauzito mwingine?* **[Outcome 3c]**

0) No 1) Yes

40. Will you attend for ANC in the same facility like last pregnancy? **[ACCEPT]**
Je utahudhuria kliniki ile ile uliyopata huduma katika ujauzito wako wa mwisho?

0) No 1) Yes

Give reason for your answer *Fafanua*……..…………………………………………………………………

41. Where would you like to **deliver** your baby in the next pregnancy? *Ungependa kujifungulia wapi iwapo utajaliwa ujauzito mwingine?*……………………………………………………………………………………

Give reason for your answer *Fafanua*……………………………………………**[ACCEPT], [Outcome 3a]**

42. If given a choice, which health facility would you use for delivery of your next baby? **[UTILIZE]** *Ingekuwa ni chaguo lako, je ungependa kuzalia kituo kipi/ au hospitali ipi na kwanini?*

Name and reason *(Jina na sababu)* ……………………………………………………………………………….

………
....

43. Will you use the health facility near to you **mentioned in Q 12a** for delivery of your next baby? *Je ukijaliwa ujauzito mwingine utatumia kituo cha karibu na wewe kujifungulia? Sababu?* **[ACCEPT]**

0) No 1) Yes

Give reason for your answer *Fafanua*……..…………………………………………………………………

44. For those who delivered at health facility, will you use the facility you used for the delivery of your last baby? *Kwa wale waliojifungulia hospitali/kituo cha afya katika ujauzito wa mwisho, je ungependa kutumia kituo/hospitali ile ile au utabadili? Sababu?* **[ACCEPT]**

0) No 1) Yes

Give reason for your answer *Fafanua*……………………………………………………………………………

45. Will you attend for **postnatal care** within 2 days after delivery of your next baby? *Je ukijaliwa kupata mtoto mwingine utaweza kwenda kupata huduma za afya ndani ya siku mbili baada ya kujifungua?*

0) No 1) Yes **[ACCEPT]**

Give reason for your answer *Fafanua*……………………………………………………………………………

46. If given a choice, which health facility would you like to use for postnatal care after delivery of your next baby? *Kama ingekuwa chaguo lako, je ungependa kuhudhuria kliniki ipi baada ya mtoto kuzaliwa iwapo utajaliwa mtoto mwingine?* **[ACCEPT]**

Name and reason *(Jina na sababu)* ……………………………………………………………………………………..
………
....

47. Will you use the health facility near to you for postnatal care for your next baby? *Je ungependa kutumia kituo kilicho karibu na kwako kwa huduma ya mama na mtoto baada ya kujifungua iwapo utajaliwa mtoto mwingine?* **[ACCEPT]**

0) No 1) Yes

Give reason for your answer *Fafanua*……………………………………………………………………………

48. In general, will you advice women to deliver at health facilities? *Kwa mtazamo wako, unashauri akina mama katika eneo hili wajifungulie katika kliniki au hospitali? Sababu?* **[ACCEPT]**

0) No 1) Yes

Give reason for your answer *Fafanua*……………………………………………………………………………

Ⅳ. Breastfeeding after last born delivery

49. Did you start breastfeeding within 1 hour after the last born delivery? **[EFFECT], [Outcome 2f]**

0) No 1) Yes

50. Did you do exclusively breastfeeding for 6 months after delivery? **[EFFECT], [Outcome 2j]**

0) No 1) Yes

51. How long after delivery did you start breastfeeding the baby? *Je ulianza kumyonyesha mtoto wa mwisho muda gani baada ya kuzaliwa?* ....hours *masaa* **[EFFECT]**

52. Frequency of breastfeeding per day for those with children below 6 months? **[EFFECT]**
Mtoto ananyonya mara ngapi kwa siku? ....(correct answer, on demand)

V. Support by family to use health facilities for MNCH services

Msaada, sapoti na ushirikiano wa familia kumuwezesha mama mjamzito kutumia vituo vya afya kwa huduma

53. Regarding your partner (*Je mume au mwenzi wako (tiki jibu atalosema);* **[ACCEPT]**

	**Specific questions**	**0. No**	**1.Yes**
A	Does he think it is important for a pregnant woman to attend at health facility for antenatal care? **[Outcome 3e]**<br>*Anaamini ni muhimu kwa mama mjamzito kuhudhuria kliniki?*		
B	Did he allow you to attend for ANC in the last pregnancy? **[Outcome 3b]**<br>*Alikuruhusu kuhudhuria kliniki katika ujauzito wako wa mwisho?*		
C	Does he believe a woman should deliver at home?<br>*Anaamini ni vizuri mama akijifungulia nyumbani?*		
D	Does he believe a woman should deliver at health facility?<br>*Anaamini ni vizuri mama akijifungulia katika hospitali au kituo cha afya?*		

54. Regarding your mother in law; *Je mama mkwe wako* **[ACCEPT]**

	**Specific questions**	**0. No**	**1.Yes**
A	Do you live with your mother-in-law at your home?<br>*Je mnaishi nyumba moja na mama mkwe?*		

B	Does she think or believe it is important for a pregnant woman to attend for antenatal care at the health facility? **[Outcome 3e]**<br>*Anaamini ni muhimu kwa mama mjazito kuhudhuria kliniki akiwa mjamzito?*		
C	Did she allow you to attend for ANC in the last pregnancy? **[Outcome 3b]**		
D	Does she believe a woman should deliver at home?<br>*Anaamini ni vizuri mama akijifungulia nyumbani?*		
E	Does she believe a woman should deliver at health facility?<br>*Anaamini ni vizuri mama akijifungulia katika hospitali au kituo cha afya?*		

55. Regarding your father in law; *Je baba mkwe wako;* **[ACCEPT]**

	**Specific questions**	**0. No**	**1.Yes**
A	Do you live with your father-in-law at your home?<br>*Je mnaishi na baba mkwe nyumba moja?*		
B	Does he think or believe it is important for a pregnant woman to attend for antenatal care at the health facility?<br>*Anaamini ni muhimu kwa mama mjazito kuhudhuria kliniki akiwa mjamzito?*		
C	Did he allow you to attend for ANC in the last pregnancy?		
D	Does he believe a woman should deliver at home?<br>*Anaamini ni vizuri mama akijifungulia nyumbani?*		
E	Does he believe a woman should deliver at health facility?<br>*Anaamini ni vizuri mama akijifungulia katika hospitali au kituo cha afya?*		

56. Will family support you to deliver at health facility if you will have another pregnancy? **[ACCESS]**
Je baba mkwe wako ni mtu anayeweza kukusaidia na kukupa ushirikiano ili ujifungulie katika kituo cha afya katika ujauzito ujao? Kama ndiyo, atakuasaidia vipi? Elezea

0) No 1) Yes

57. General in your cultural beliefs, how does the community perceive a woman who delivers at the hospital or health facility? **[ACCEPT]**
Je kwa ujumla, katika mila na desturi zenu, jamii yenu inamchukuliaje mama aliyejifungulia katika kituo cha ya afya?

68. Again in your opinion what key things should be done or improved so that women would be able and would like to deliver at health facilities? **[ACCESS]**

Kwa ushauri wako ni nini kifanyike ili akina mama waweze na wapende kujifungulia katika vituo vya afya?

Upande wa serikali? Upande wa kliniki zenyewe? Upande wa jamii? Upande wa familia?

We have come to the end of questionnaire. *Mwisho wa mahojiano.*

Do you have any questions or any concern concerning the services given during pregnancy, childbirth or during postpartum period which you would like to discuss?

Record key concerns to discuss with health facility or district administration:
