# Supplementary material for: Bottlenecks Analysis in the Intervention of Improving Maternal Health in Rural Areas of Tanzania: A Convergent Mixed-Method Approach
Source: Int J Health Policy Manag. 2025 Mar 2;14:8355. doi: 10.34172/ijhpm.8355 (PMC12032231; doi:10.34172/ijhpm.8355)
Supplement: Supplementary file 2 — Calculate Sample Size for Survey of Women in Reproductive Age. [file ijhpm-14-8355-s002.pdf]

Article title: Bottlenecks Analysis in the Intervention of Improving Maternal Health in Rural Areas of Tanzania: A Convergent Mixed-Method Approach
Journal name: International Journal of Health Policy and Management (IJHPM)
Authors' information: Hyeyun Kim[1,2], Jiye Kim[2], Seohyeon Lee[2], Minkang Cho[2], Hyekyeong Kim[3]*
[1]Korea Institute for Health and Social Affairs, Sejong, Republic of Korea.
[2]Department of Health Convergence, Graduate School of Ewha Womans University, Seoul, Republic of Korea.
[3]Department of Health Convergence, Ewha Womans University, Seoul, Republic of Korea.
***Correspondence to:** Hyekyeong Kim; Email: hkkim@ewha.ac.kr
Citation: Kim H, Kim J, Lee S, Cho M, Kim H. Bottlenecks analysis in the intervention of improving maternal health in rural areas of Tanzania: a convergent mixed-method approach. Int J Health Policy Manag. 2025;14:8355.doi:10.34172/ijhpm.8355
Supplementary file 2. Calculate Sample Size for Survey of Women in Reproductive Age

The sample size was calculated based on the formula below (i.e. multiple indicator cluster survey formula)

$$n = \frac{[\ 4\ (r)\ (1\text{-}r)\ (f)\ (1.1)\ ]}{[\ (0.12r)^2\ (p)\ (n_h)\ ]}$$

Where

- **n** is the required sample size, expressed as number of households, for the KEY indicator (see following section on determining the key indicator)
- **4** is a factor to achieve the 95 per cent level of confidence
- **r** is the predicted or anticipated prevalence (coverage rate) for the indicator being estimated
- **1.1** is the factor necessary to raise the sample size by 10 per cent for non-response
- **f** is the shortened symbol for *deff* (i.e. design effect) = 2.0
- **0.12r** is the margin of error to be tolerated at the 95 per cent level of confidence, defined as 12 per cent of r (12 per cent thus represents the relative sampling error of r)
- **p** is the proportion of the total population upon which the indicator, r, is based (i.e. women of reproductive age: 25 % (Census, 2013)
- **n_h** is the average household size (i.e.=4 by Census Report, 2013)

Cluster size was be set at 30

Effective number of household was the "Number of households (Sample Size) /0.9"

By plugging the value into the formula above

N (sample size) = $4x0.20x0.80x2.0x1.1/(0.12x0.2)^2*0.25*4.9$ = 1,657 households per district.

Effective number of households = 1,657 /0.9= **1,841 HH**

Therefore, the minimum sample size of women of reproductive age required ˜ 390

Enumeration areas needed:

Number of enumeration area in the region 61EA (=1,841/30)

Then, allocating 61 EA to 5 districts with an average of 13 EA depending on the % of women per each district gives the sample size for each district as shown in Table 2.3 below.

Table S1: Procedure for sample estimation

District	Number of households (Sample Size)	Effective number of households (#HH/0.9)	cluster	(Effective number of households/cluster) EAs	EAs (rounded)	Sample size per district (EA*30)
Kishapu	402	447	30	13.4	13	390
