# Supplementary material for: Bottlenecks Analysis in the Intervention of Improving Maternal Health in Rural Areas of Tanzania: A Convergent Mixed-Method Approach
Source: Int J Health Policy Manag. 2025 Mar 2;14:8355. doi: 10.34172/ijhpm.8355 (PMC12032231; doi:10.34172/ijhpm.8355)
Supplement: Supplementary file 3 — General Characteristics of Quantitative and Qualitative Study Participants. [file ijhpm-14-8355-s003.pdf]

Article title: Bottlenecks Analysis in the Intervention of Improving Maternal Health in Rural Areas of Tanzania: A Convergent Mixed-Method Approach

Journal name: International Journal of Health Policy and Management (IJHPM)

Authors' information: Hyeyun Kim[1,2], Jiye Kim[2], Seohyeon Lee[2], Minkang Cho[2], Hyekyeong Kim[3]*

[1]Korea Institute for Health and Social Affairs, Sejong, Republic of Korea.

[2]Department of Health Convergence, Graduate School of Ewha Womans University, Seoul, Republic of Korea.

[3]Department of Health Convergence, Ewha Womans University, Seoul, Republic of Korea.

***Correspondence to:** Hyekyeong Kim; Email: hkkim@ewha.ac.kr

Citation: Kim H, Kim J, Lee S, Cho M, Kim H. Bottlenecks analysis in the intervention of improving maternal health in rural areas of Tanzania: a convergent mixed-method approach. Int J Health Policy Manag. 2025;14:8355.doi:10.34172/ijhpm.8355

Supplementary file 3. General Characteristics of Quantitative and Qualitative Study Participants

Table S3-1: General characteristics of quantitative study participants

Data collection methods / Target	Characteristics		N	%
Survey / Women in reproductive age		Total	400	100.0
	Age group	13-24	172	43.0
		25-34	169	42.3
		35-44	55	13.8
		45+	4	1.0
	Marital status	Married	289	72.3
		Cohabiting	74	18.5
		Single	26	6.5
		Divorce/Separated	10	2.5
		Widow	1	0.3
	Education level	Never been to school	30	7.5
		Primary	321	80.3
		Secondary	48	12.0
		Tertiary	1	0.3
Health facility assessment / Health facility		Total	49	100.0
	Type of health facility	Hospital	1	2.0
		Health center	3	6.2
		Dispensary	45	91.8

Table S3-2: General characteristics of qualitative study participants

Data collection methods	Target	Region	Number of participants	Average age	Average number of family members
IDI	Women in reproductive age	Total	20	27.9	7
		Mipa	8	25.6	6
		Illebelebe	12	29.6	7
FGD	Women in reproductive age	Total	21	29.9	8
		Mipa	13	30.5	8
		Illebelebe	8	29.4	8
	Husbands	Total	10	36.2	7
		Mipa	5	39.6	8
		Illebelebe	5	32.8	5
	Mothers-in-law	Total	10	50.2	8
		Mipa	5	51.8	5
		Illebelebe	5	48.6	11
**Data collection methods**	**Target**	**Region**	**Number of participants**	**Average age**	**Year of work experience**
FGD	Community health worker	Total	10	39.0	9.6
		Mipa	5	40.8	9.8
		Illebelebe	5	37.2	9.4
	Health professional	Total	5	43.2	10.1
		Mipa	3	42.7	10.2
		Illebelebe	2	44.0	10.0
