# Supplementary material for: Bottlenecks Analysis in the Intervention of Improving Maternal Health in Rural Areas of Tanzania: A Convergent Mixed-Method Approach
Source: Int J Health Policy Manag. 2025 Mar 2;14:8355. doi: 10.34172/ijhpm.8355 (PMC12032231; doi:10.34172/ijhpm.8355)
Supplement: Supplementary file 4 — Focus Group Discussion Guide. [file ijhpm-14-8355-s004.pdf]

Article title: Bottlenecks Analysis in the Intervention of Improving Maternal Health in Rural Areas of Tanzania: A Convergent Mixed-Method Approach
Journal name: International Journal of Health Policy and Management (IJHPM)
Authors' information: Hyeyun Kim[1,2], Jiye Kim[2], Seohyeon Lee[2], Minkang Cho[2], Hyekyeong Kim[3]*
[1]Korea Institute for Health and Social Affairs, Sejong, Republic of Korea.
[2]Department of Health Convergence, Graduate School of Ewha Womans University, Seoul, Republic of Korea.
[3]Department of Health Convergence, Ewha Womans University, Seoul, Republic of Korea.
***Correspondence to:** Hyekyeong Kim; Email: hkkim@ewha.ac.kr
Citation: Kim H, Kim J, Lee S, Cho M, Kim H. Bottlenecks analysis in the intervention of improving maternal health in rural areas of Tanzania: a convergent mixed-method approach. Int J Health Policy Manag. 2025;14:8355.doi:10.34172/ijhpm.8355
Supplementary file 4. Focus Group Discussion Guide

Ⅰ. General Guideline for Focus Group Discussion

1. Moderator Guide

■ Use Purposeful small talk (Rapport)

- Intro talk should be related with research problem
- Include topics about living area (distance from major institution), family members, medical interest etc.

■ Make a smooth introduction

- Welcome address, Overview of topic, Ground rules, first question

■ Use pauses and probes

- 5-6 second pause (wait for response)
- Probes: Would you explain further?, Would you give an example?, I don't understand etc.

■ Control reaction to participants

- Verbal and nonverbal, Head nodding, Short verbal response
- Avoid judgement (ex. That is good, Excellent etc.)

■ Use appropriate conclusion

- Summarize with confirmation, review purpose and ask if anything has been misses, thanks and dismissal

■ Use name card for participants

- Place the name cards on the table in front of participants to help remember each other's name

2. Consent Process Guide

Consent forms for focus group participants are completed in advance by all those seeking to participate. Below is a summary of the information in the consent form that focus group organizers and facilitators should use to make sure participants understand the information in the consent form.

Thank you for agreeing to participate. We are very interested to hear your valuable opinion on maternal health and utilization of related services in your community.

- *The purpose of this study is to learn difficulties to utilize MH services at a health facility. We hope to learn things that how health facilities and project team can contribute to improve your MH health in your home, village, and Tanzania.*
- *The information you give us is completely confidential, and we will not associate your name with anything you say in the focus group.*
- *We would like to tape the focus group so that we can make sure to capture your thoughts, opinions, and ideas we hear from the group. No names will be attached in the analysis and the records will be destroyed as soon as they are transcribed.*
- *You may refuse to answer any question or withdraw from the study at any time.*
- *We understand how important it is that this information is kept private and confidential. We will ask participants to respect each other's confidentiality.*
- *If you have any questions now or after you have completed the questionnaire*, you can always contact a study team member like me, or you can contact the Tanzania research team leaders whose names and contact are on consent form. *Brief questions will be provided to participants*
- *Please check the consent form and sign to show you agree to participate in this focus group.*

3. Introduction Guide

■ Step 1. Welcome: Introduce moderator and related staffs

■ Step 2. Topic introduction and consent

Do not limited topic as maternal health too much. Various story resources could be inspired to researcher and provide ideas

■ Step 3. Guidelines (Rules)

- No right or wrong answers, only differing points of view
- We are on recording, one person speaking at a time
- We are on a first name basis
- You don't need to agree with others but please listen respectfully as others share their life and views
- We ask that you turn off your phone. If you cannot please put your phone on vibrate. You cannot answer the phone in here, if you must respond to a call, please do it outside and rejoin us as quickly as you can.
- My role as moderator will be to guide the discussion
- Talk to each other

■ Step 4. Opening Question

4. Asking Questions for useful information

■ Use open-ended questions

- e.g. What did you think of relationship between your mother and wife?

  How did you feel about your wife's health condition during pregnancy?

  Where do you get information about pregnant woman and newborns?

■ Avoid dichotomous questions

■ Rare use asking reason – Why question

- Instead ask about attributes and/or influences. Attributes are characteristics or features of the topic. Influences are things that prompt or cause action

■ Use "think back" questions

- Take people back to an experience and not forward to the future

■ Use different types of questions

- Opening question – Introductory question – Transition question – Key question – Ending question

■ Use questions that get participants involved

■ Focus the questions

■ Be caution of serendipitous questions

Ⅱ. Focus Group: Woman related with ANC

: Who received ANC 0 or 1 time, but did not receive prenatal care within 12 weeks

1. Main issues

- Difficult to access health facilities and accept their services
- Low degree of services utilization
- Low quality of services at a health facility

2. Purpose of focus group discussion

(1) Understand mothers' attitudes toward pregnancy, delivery, pre/postnatal care

(2) Understand main barriers for mothers to use 4 times prenatal services and start the 1st one before 12th week

(3) Check family support of utilization of health facility and its MNCH services

(4) Figure out the degree of understanding on RMNCH and its information resource

(5) Whether women feel the necessity of MNCH services

(6) Figure out accessibility to MNCH services and health facility

(7) Awareness/satisfaction/usefulness of Good Neighbors projects and its awareness change activities

3. Composition of Questions

Component	Main indicators	Contents
Awareness and support from family and community	Accessibility, Acceptability	• Attitudes toward pregnancy/delivery within household and community<br>• Problems in current health facility and MNCH services<br>• Decision maker within household/ Financial burden in general issues and health<br>• Mother-in-law/husband's acceptance (complaint or concerns, worries, hindrances, etc) of health facility and MNCH services<br>• Overall pattern of bringing up children<br>• Expectation of supportive action from family members
Opportunity for acquisition of knowledge	Acceptability, Utilization, Effectiveness	• Opportunity to acquire RMNCH knowledge<br>• Reliability of knowledge<br>• Resource of knowledge about pregnancy/ delivery

Willingness to act	Acceptability, Utilization, Effectiveness	• Consensus on MNCH knowledge<br>• MNCH knowledge vs. traditional ones<br>• Actual contribution to one's own health and life
Accessibility to health facility	Accessibility, Utilization	• Accessibility to health facility<br>• Satisfaction of MNCH services
Evaluation of recent MNCH activities	Overall evaluation	• Awareness/satisfaction of Good Neighbors activities<br>• Awareness/satisfaction of awareness change activities<br>* *Awareness/satisfaction of photovoice activity – Only Mipa dispensary and its catchment areas*

Ⅲ. Focus Group: Women related with PNC

: Who received ANC 3-4 times, but did not receive postnatal care

1. Main issues

- Low rate of facility delivery that leads to low rate of receiving postnatal care
- Difficult to utilize PNC (Mothers' lack of knowledge and understanding of the importance of PNC)

2. Purpose of focus group discussion

(1) Understand mothers' attitudes and knowledge of pregnancy/ delivery

(2) Understand the reasons/barriers for not using postnatal care.

(3) Understand mothers' attitudes toward postnatal care, MNCH services and health facility

(4) Check family support of utilization of health facility and its MNCH services

(5) Figure out the degree of mother's understanding on RMNCH and its information resource

(6) Whether women feel the necessity of MNCH services

(7) Satisfaction of MNCH services and health facility

(8) Awareness/satisfaction/usefulness of Good Neighbors projects and its awareness change activities

3. Composition of Questions

Component	Main indicators	Contents
Awareness and support from family and community	Accessibility, Acceptability	• Attitude to pregnancy/ delivery within household and community<br>• Support level of health facility and MNCH services<br>• Decision maker within household for general issues and health issues/ Financial burden<br>• Mother-in-law/ husband's acceptance of health facility and MNCH services<br>• Overall pattern of bringing up children<br>• Expectation of supportive action from family
Opportunity to acquisition of knowledge	Acceptability, Utilization, Effectiveness	• Opportunity to acquisition of RMNCH knowledge<br>• Reliability of knowledge<br>• Resource of knowledge about pregnancy/ delivery
Willingness to act	Acceptability, Utilization, Effectiveness	• Consensus on MNCH knowledge<br>• MNCH knowledge vs. traditional ones<br>• Actual contribution to one's own health and life
Satisfaction of health facility	Accessibility, Utilization	• Satisfaction of health facility/staff/health volunteers<br>• Satisfaction of MNCH service experiences
Evaluation of recent MNCH activities	Overall evaluation	• Awareness/satisfaction of Good Neighbors activities<br>• Awareness/satisfaction of awareness change activities<br>* *Awareness/satisfaction of photovoice activity – Only Mipa dispensary and its catchment areas*

Ⅳ. Focus Group: Husbands

: Whose wife who received ANC 0 or 1 time, but did not receive prenatal care within 12 weeks *OR* ANC 3-4 times, but did not receive postnatal care

1. Main issues

- Low geographic and psychological accessibility
- Low degree of support on health facility utilization
- Trustiness of health facility and its services

2. Purpose of focus group discussion

(1) Understand the main barriers for delivery, pre/postnatal care at health facilities

(2) Understand the level of acceptance/ preference of MNCH services at health facilities

(3) Understand utilization pattern of health facility and its accessibility and trustiness

(4) Check general level of understanding and support of MNCH within men(husband)

(5) Awareness/satisfaction/usefulness of Good Neighbors projects and its awareness change activities

3. Composition of Questions

Component	Main indicators	Contents
General	Accessibility, Acceptability (Socio-cultural factor)	• General lifestyle and environment<br>• Socio-cultural factor with religion and practices
Utilization pattern of health facility	Accessibility, Utilization	• Frequency of health facility utilization<br>• Psychological intimacy and trustiness of health facility<br>• Financial burden
Interest in home life	Accessibility, Acceptability	• Interest in relationship within family<br>• Interest in wife and children<br>• Power of decision making
Knowledge and interest in MNCH	Utilization, Effectiveness	• Knowledge and interest in MNCH<br>• Interest or participation in neonatal and child health<br>• Acceptance/ preference of MNCH<br>• Attitudes toward pregnancy, birth, pre/postnatal care
Evaluation of recent MNCH activities	Overall evaluation	• Awareness/satisfaction of Good Neighbors activities<br>• Awareness/satisfaction of awareness change activities<br>** Awareness/satisfaction of photovoice activity – Only Mipa dispensary and its catchment areas*

Ⅴ. Focus Group: Mothers-in-Law

: Whose daughter-in-law who received ANC 0 or 1 time, but did not receive prenatal care within 12 weeks *OR* ANC 3-4 times, but did not receive postnatal care

1. Main issues

- Balance of decision making within household on general issues and health issues
- Low degree of support on health facility utilization
- Trustiness of health facility and its services

2. Purpose of focus group discussion

(1) Understand relationship between family members and balance of decision making

(2) Figure out general understanding/ acceptance/ preference of new MNCH which is different from their own experiences

(3) Understand the main barriers for delivery, pre/postnatal care at health facilities

(4) Understand the level of acceptance/ preference of MNCH services at health facilities

(5) Understand utilization pattern of health facility and its accessibility and trustiness

(6) Check general level of understanding and support of MNCH within older women and other community residents

(7) Awareness/satisfaction/usefulness of Good Neighbors projects and its awareness change activities

3. Composition of Questions

Component	Main indicators	Contents
General	Accessibility, Acceptability (Socio-cultural factor)	• General lifestyle and environment<br>• Socio-cultural factor with religion and practices<br>• Overall behavior pattern of MNCH within community
Utilization pattern of health facility	Accessibility, Utilization	• Attitudes toward health maintenance<br>• Attitudes and their own experiences toward pregnancy, birth, pre/postnatal care<br>• Frequency of health facility utilization<br>• Psychological intimacy and trustiness of health facility<br>• Financial burden
Relationship between family members	Accessibility Acceptability	• Relationship between family member<br>• Decision maker<br>• Relationship between male and female
Evaluation of recent MNCH activities	Overall evaluation	• Awareness/satisfaction of Good Neighbors activities<br>• Awareness/satisfaction of awareness change activities<br>** Awareness/satisfaction of photovoice activity – Only Mipa dispensary and its catchment areas*

Ⅵ. Focus Group: Health Facility Workers

1. Main issues

- Availability to provide health services and operate health facility
- Willingness and behavior to provide higher quality of health services

2. Purpose of focus group discussion

(1) Do SWOT analysis on health facility and community related with MNCH

(2) Understand community health issue and its priority

(3) Figure out operation level of health facility and quality of service provision

(4) Figure out willingness and actual behavior to improve MNCH in community

(5) Awareness/Experiences of Good Neighbors project and its activities

(6) Their suggestions for improving Good Neighbors project and its activities

3. Composition of Questions

Component	Main indicators	Contents
Characteristics of health facility	Availability, Utilization, Effectiveness	• SWOT analysis on health facility and community
Community health issue	Acceptability	• Awareness and acceptance of MNCH in community<br>• Community health issue and its priority
Operation and services provision	Availability	• Fidelity of operation of health facility (operation time, staffing)<br>• Availability of medicine and equipment<br>• Scope of ANC/ PNC/ delivery to provide
Evaluation of recent MNCH activities	Overall evaluation	• Awareness/satisfaction of Good Neighbors activities<br>• Awareness/satisfaction of awareness change activities<br>** Awareness/satisfaction of photovoice activity – Only Mipa dispensary and its catchment areas*<br>• Suggestion for future improvement

Ⅶ. Focus Group: CHW

1. Main issues

- Low utilization of prenatal and postnatal care services
- Low acceptability of community on MNCH related education and activities

2. Purpose of focus group discussion

(1) Check CHW's understanding of MNCH projects

(2) Check CHW's understanding of provision scope of MNCH services

(3) Check CHW's willingness to improve MNCH and its barriers

(4) Check CHW's expertise of MNCH activities

(5) Check CHW's understanding of the main barriers for delivery, pre/postnatal care at health facilities in the community

(6) Check CHW's understanding of the level of acceptance/ preference of MNCH services at health facilities

(7) Check CHW's understanding of mothers' utilization pattern of health facility and its accessibility and trustiness

(8) Awareness/satisfaction/usefulness of Good Neighbors projects and its awareness change activities

3. Composition of Questions

Component	Main indicators	Contents
Characteristics of community	Acceptability, Utilization	• Analysis on health facility and community related with MNCH health
MNCH services	Availability, Utilization	• Provision scope of MNCH services<br>• Education and monitoring system to train CHW
Willingness of improving MNCH	Utilization, Effectiveness	• Willingness and actual behavior of improving MNCH<br>• Barriers to activity
Evaluation of recent MNCH activities	Overall evaluation	• Awareness/satisfaction of Good Neighbors activities<br>• Awareness/satisfaction of awareness change activities<br>* *Awareness/satisfaction of photovoice activity*

		– Only Mipa dispensary and its catchment areas
