# Supplementary material for: Bottlenecks Analysis in the Intervention of Improving Maternal Health in Rural Areas of Tanzania: A Convergent Mixed-Method Approach
Source: Int J Health Policy Manag. 2025 Mar 2;14:8355. doi: 10.34172/ijhpm.8355 (PMC12032231; doi:10.34172/ijhpm.8355)
Supplement: Supplementary file 5 — In-Depth Interview Guide. [file ijhpm-14-8355-s005.pdf]

Article title: Bottlenecks Analysis in the Intervention of Improving Maternal Health in Rural Areas of Tanzania: A Convergent Mixed-Method Approach

Journal name: International Journal of Health Policy and Management (IJHPM)

Authors' information: Hyeyun Kim[1,2], Jiye Kim[2], Seohyeon Lee[2], Minkang Cho[2], Hyekyeong Kim[3]*

[1]Korea Institute for Health and Social Affairs, Sejong, Republic of Korea.

[2]Department of Health Convergence, Graduate School of Ewha Womans University, Seoul, Republic of Korea.

[3]Department of Health Convergence, Ewha Womans University, Seoul, Republic of Korea.

***Correspondence to:** Hyekyeong Kim; Email: hkkim@ewha.ac.kr

Citation: Kim H, Kim J, Lee S, Cho M, Kim H. Bottlenecks analysis in the intervention of improving maternal health in rural areas of Tanzania: a convergent mixed-method approach. Int J Health Policy Manag. 2025;14:8355.doi:10.34172/ijhpm.8355

Supplementary file 5. In-Depth Interview Guide

Ⅰ. General guideline for In-depth interview

This study will use 'storytelling' methods for IDI. In the qualitative research, gathering a variety of stories helps to understand complex, interconnected contexts related to research. General guideline of storytelling is as follows

1. Composition of storytelling

(1) Start from general stories and move to personal details

(2) Composition of pregnancy related storyline over time: From the time of recognize the pregnancy until postnatal care

(3) Interview place should be a familiar place to the participant.

2. Procedure

- The storytelling method proceeds by sharing stories between interviewer and interviewees. First, interviewer should provide the story. Give a brief case story consisting of the information that wants to know from the participants. It is most effective when it includes one or two main contents – less than three. The focusing topic (what you are most interested) is mainly placed at the end with negative case.
  - However, if participant answer to the question with shorts answers like "Same as you" or "Similar on you", be sure to get the answer with specific experiences of the participants
  - Construct a consistent flow by pre-organizing the whole story (which is provide to participants)

- Provided stories (examples) should be consisted of similar conditions with interviewee's own condition.

- Listen to the interviewee's experiences – Make sure that all the elements you want to get are included
- Extend the stories according to the stages (Stages are provided in the next section)
- Rather than repeating patterns, use proper and refreshing transition questions

3. Stages of Storytelling for IDI

Storytelling will be divided into 4 stages according to time flow and general issues to personal issues.

Stage	Main Contents
General details	General life pattern, Family background, Accessibility to Health Facility, Family's support for pregnancy and delivery, Level of Family's support/ acceptance for MNCH services
Recognition of Pregnancy + Antenatal Care	How to recognize the pregnancy, Whether or not one utilized antenatal care services, Level of Satisfaction of services, Barriers to receive antenatal care services, Knowledge of MNCH
Delivery	Where to deliver (Health Facility/Home), Situation at the delivery, Personal feeling
Postnatal Care	Whether or not one utilized postnatal care services, Satisfaction of services, Barriers to receive postnatal care services

Ⅱ. Details by Target Interviewees

1. Target Interviewee - Women related with ANC (total 5 women)

1) 2 women who had deliveries within last 3 years but did not receive ANC (but who had experiences of health facility visits)

Target is women who does not show any changes despite of project intervention

▶ Accessibility/Acceptability-focused Story telling

2) A woman who received 1 time of ANC, but not within 12 weeks

Target is a woman who had the will to receive ANC, but also barriers to receiving the service at the right time

▶ Accessibility/Effectiveness-focused Story telling

3) A woman who received 3 times of ANC within 12 weeks after project intervention while she received 0-1 times of ANC before project intervention

Target is a woman significantly changed her performance

▶ Accessibility/Effectiveness-focused Story telling

4) A women who received 2-3 times of ANC after project intervention while she did not receive ANC before project intervention

Target is women who significantly changed her behaviors

▶ Accessibility/Acceptability/Utilization-focused Story telling

2. Target Interviewee- Women related with PNC (total 5 women)

1) A woman who had the will to have a postnatal care at health facility, but could not be received (due to financial burden, opposition by family or due to other reasons)

   ▶ Accessibility/Utilization-focused Story telling

2) A woman who did not deliver at health facility but received PNC within 7 days

   ▶ Accessibility/Acceptability/Effectiveness-focused Story telling

3) A woman who delivered at health facility but did not receive PNC within 48 hours

   ▶ Acceptability/Utilization-focused Story telling

4) 2 women who received traditional PNC (local customs of belief), but did not receive PNC at health facility

   ▶ Accessibility/Acceptability-focused Story telling

3. Main Questions by Each Stage

Stage	Main Observation Theme	Contents
General Details	Living Environment & Sociocultural Acceptability	· Recognition of MNCH service within the household/community<br>· Family members' attitudes towards/ knowledge of MNCH<br>· Financial Status<br>· Relationship b/w family members
	Health Facility Utilization	· Accessibility to, confidence in, and intimacy with Health facility<br>· Level of Family members' utilization of Health facility
Recognition of Pregnancy + ANC	Knowledge of MNCH	· Chance to acquire MNCH knowledge<br>· Changes in awareness and acceptance of the significance of MNCH before/after the pregnancy (intervention?)<br>· Changes in behavior and willingness for MNCH service by knowledge acquisition

	Barriers to Service Utilization	· Family member's response to the pregnancy<br>· Family members' support behavior during the pregnancy<br>· Level of Satisfaction of ANC services<br>· Changes in their life pattern with pregnancy<br>· Other issues during recent pregnancy
Delivery	Situation at the delivery	· Delivery at Health facility/home & reason for delivery place<br>· Family member's response to the delivery<br>· Change in life pattern by the delivery
	Personal feeling	· Feeling about the delivery<br>· Feeling about family members' response to the delivery<br>· Feeling about the MNCH service
PNC	Barriers to Service Utilization	· Family members' recognition of PNC<br>· Family members' support behavior for PNC<br>· Change in life patter after delivery<br>· Level of Satisfaction of PNC service<br>· Knowledge of PNC<br>· Other barriers to getting PNC
	Willingness and actual behavior of improving MNCH	· Personal feeling during the period of pregnancy-delivery<br>· Personal feeling about family members during the period of pregnancy-delivery (including postnatal period)<br>· Expectations for family members<br>· Expectations for Health facility<br>· What one think is the most significant during the period of pregnancy-delivery
