# Supplementary material for: Bottlenecks Analysis in the Intervention of Improving Maternal Health in Rural Areas of Tanzania: A Convergent Mixed-Method Approach
Source: Int J Health Policy Manag. 2025 Mar 2;14:8355. doi: 10.34172/ijhpm.8355 (PMC12032231; doi:10.34172/ijhpm.8355)
Supplement: Supplementary file 6 — Theme-Code Structure of Thematic Analysis. [file ijhpm-14-8355-s006.pdf]

Article title: Bottlenecks Analysis in the Intervention of Improving Maternal Health in Rural Areas of Tanzania: A Convergent Mixed-Method Approach

Journal name: International Journal of Health Policy and Management (IJHPM)

Authors' information: Hyeyun Kim[1,2], Jiye Kim[2], Seohyeon Lee[2], Minkang Cho[2], Hyekyeong Kim[3]*

[1]Korea Institute for Health and Social Affairs, Sejong, Republic of Korea.

[2]Department of Health Convergence, Graduate School of Ewha Womans University, Seoul, Republic of Korea.

[3]Department of Health Convergence, Ewha Womans University, Seoul, Republic of Korea.

***Correspondence to:** Hyekyeong Kim; Email: hkkim@ewha.ac.kr

Citation: Kim H, Kim J, Lee S, Cho M, Kim H. Bottlenecks analysis in the intervention of improving maternal health in rural areas of Tanzania: a convergent mixed-method approach. Int J Health Policy Manag. 2025;14:8355.doi:10.34172/ijhpm.8355

Supplementary file 6. Theme-Code Structure of Thematic Analysis.

Theme	Code	Sub-code
Availability	Scope of Services (ANC, delivery and PNC)	
	Availability of equipment and supplies	
	HF Staffing	
Accessibility	Accessibility to health facility	
	Accessibility of Ante Natal and Labour and Delivery Care Services	
	Accessibility of Post Natal Care	
	Family support in Accessing ANC services	
	Cultural/customs/Taboos in accessing ANC and Labour and Delivery services	
Acceptability	Knowledge of MH services	
	Perceived Necessity of MH services	
	Reliability of the knowledge received	
	Mothers' and husbands' attitudes toward MH services	
	Community Engagement	
	Awareness or satisfaction of Good Neighbours' projects and its awareness change activities	
Utilization	Pattern of family support on utilization of health facility and its MH services	Frequency of HF utilization by husbands
		Decision making within household with regard to health in general
	Utilization pattern of MNCH services	Ante Natal care (Visits 4 times and 1st on 12th week)
		Delivery at the health facility
		Post Natal Care
		Barriers for CHW activities
Quality of MH Services	Quality of MH services provided at the facility	
	Quality of MH services provided by Health Care Workers (HCW)	Scope of ANC and PNC education provided
		Monitoring system for CHW
	Operating time	
